# Elevated circulating fibroblast growth factor 23 is associated with acute kidney injury and mortality in coronavirus disease 2019 (COVID-19)

**DOI:** 10.1590/2175-8239-JBN-2025-0377en

**Published:** 2026-08-24

**Authors:** Gayatri Narayanan, Monique Opuszcka Campos, Nolan Groninger, Heather N. Burney, Matthew Hays, Matthew Dollins, Eliott Arroyo, Arvin Halim, Yang Li, Syed Jawad Sher, Sharon Moe, Kenneth Lim

**Affiliations:** 1 Indiana University School of Medicine, Department of Medicine, Division of Nephrology & Hypertension, Indianapolis, United States.; 2 Indiana University School of Medicine, Department of Biostatistics and Health Data Science, Indianapolis, United States.; 3 Wake Forest University, Department of Health and Exercise Science, Winston-Salem, United States.

**Keywords:** Acute Kidney Injury, Biomarkers, COVID-19, Fibroblast Growth Factor-23, Mortality

## Abstract

**Introduction:**

Acute kidney injury (AKI) is a key negative prognostic factor for survival in patients hospitalized with COVID-19, but few reliable biomarkers for AKI and mortality exist. This study aimed to evaluate whether elevated fibroblast growth factor 23 (FGF23) levels are associated with AKI status and mortality in COVID-19 patients.

**Methods:**

In this prospective cohort of 111 COVID-19 patients hospitalized at the Indiana University Academic Health Center (April–October 2020), circulating FGF23 and its co-receptor Klotho levels were assessed for association with AKI and 28-month survival.

**Results:**

Of the 111 patients, 77 had no AKI (91.0 [69.2, 101.3] mL/min/1.73 m^2^), 17 had AKI (39.7 [28.7, 57.8] mL/min/1.73 m^2^), and 17 had end-stage kidney disease (ESKD; 11.9 [8.6, 17.5] mL/min/1.73 m^2^). Median follow-up was 22.6 months. Median FGF23 levels were higher in patients with AKI (305.6 [134.6, 350.8] RU/mL) and ESKD (3,607.6 [440.5, 7,452.7] RU/mL) compared to those without AKI (120.7 [64.3, 249.7] RU/mL; *P* < 0.001). Patients (excluding those with ESKD) with elevated FGF23 levels had increased odds of having AKI (OR: 2.30, 95% CI: [1.31, 4.03]; *P* = 0.004). Moreover, each unit increase in log(FGF23) was associated with a 45% higher risk of mortality in the cohort (HR: 1.45, 95% CI: [1.02, 2.07]; *P* = 0.04) after controlling for age, cardiovascular disease, and BMI. Klotho levels differed by group (without AKI: 576.1 [455, 730] pg/mL; with AKI: 594.6 [416, 774] pg/mL; ESKD: 421.5 [330, 562] pg/mL; *P* = 0.04), but were not associated with AKI status.

**Conclusion:**

Elevated FGF23 is associated with AKI status and mortality in patients with COVID-19.

## INTRODUCTION

In 2019, the emergence of the infectious agent SARS-CoV-2 (COVID-19) virus led to a global pandemic that has amassed over 600 million cases and resulted in over 6.5 million deaths worldwide^1^. Since then, COVID-19 has remained the third leading cause of death from 2020 to 2022^2^. COVID-19 has a biologically versatile nature that can directly target several organ systems, including the respiratory, cardiovascular, and urinary systems. One of numerous major complications resulting from COVID-19 is acute kidney injury (AKI). AKI develops as a secondary complication in approximately 20-40% of critically ill patients with COVID-19, and up to 20% of patients with COVID-19 admitted to the intensive care unit (ICU) require renal replacement therapy (RRT)^3^. Additionally, patients with COVID-19 and resultant AKI have a significantly increased risk of in-hospital mortality compared to patients with COVID-19 but without AKI^3^. Therefore, the presence of AKI is considered to be a negative prognostic factor for survival in patients in the ICU. However, to date, there are no established early biomarkers for assessing the risk of AKI secondary to COVID-19 infection.

Fibroblast growth factor 23 (FGF23) is a bone-derived phosphatonin that rises during the early stages of renal impairment and is tightly linked to AKI and mortality in critically ill patients. Elevated FGF23 levels counteract rising phosphate levels caused by renal impairment by binding to a Klotho-fibroblast growth factor receptor 1 (FGFR1) receptor complex in the kidneys^4^. This interaction results in the suppression of the sodium-phosphate cotransporters NaPi2a and NaPi2c in the proximal tubules, suppression of the sodium-phosphate cotransporters NaPi2b in the intestine, downregulation of 1α-hydroxylase, and the upregulation of 24-hydroxylase^5^. These net effects of FGF23 include a reduction in 1,25-hydroxyvitamin D (calcitriol) levels, inhibition of intestinal phosphate absorption, and stimulation of renal phosphate wasting to help counteract hyperphosphatemia. Significantly, elevated FGF23 levels are independently associated with an increased risk of cardiovascular events, and emerging evidence suggests that elevated levels also predict in-hospital mortality in patients with AKI and chronic kidney disease (CKD)^6,7,8,9,10^.

While previous studies have examined inflammatory biomarkers and other established biomarkers of kidney injury in COVID-19^11^, to the best of our knowledge, the association between circulating FGF23 levels and AKI status and mortality outcomes in this population remains unexplored. This issue is particularly relevant due to emerging evidence that FGF23 may serve as a novel biomarker of AKI. Intriguingly, elevated FGF23 levels after AKI manifest before any alterations in classic biomarkers of kidney function, such as NGAL in mice and serum creatinine and mineral metabolites in patients after cardiac surgery and those with critical illness^6,12^. However, whether this biological signal is present and associated with clinical outcomes in the context of COVID-19 remains unknown. Therefore, studies aimed at improving the assessment of the risk of developing AKI and better estimating mortality risk in patients with COVID-19 could facilitate prompt intervention and improve AKI management strategies and patient outcomes. This is particularly important given that COVID-19 has now become endemic and continues to be a leading cause of death in vulnerable populations. Herein, we conducted a prospective cohort study to determine whether FGF23 levels are associated with AKI status and mortality in adults hospitalized with COVID-19.

## METHODS

### Study design and cohort

We conducted a prospective cohort study involving a total of 111 patients who had a positive diagnosis of COVID-19 and were hospitalized at the Indiana University Health Academic Health Center (Indianapolis, IN, USA) between April 2020 and October 2020. Inclusion criteria included hospitalization with a confirmed diagnosis of COVID-19 and patient age of ≥ 18 years. Blood samples were collected within 27 days of initial hospital admission and subsequently stored in the Indiana Biobank (Indianapolis, IN, USA). Samples and clinical data were accessed for research purposes starting on April 4, 2021. De-identified clinical data were provided by the Regenstrief Institute, Inc., a health informatics organization that provides data from the Indiana Network for Patient Care (INPC). Individuals meeting the inclusion criteria were followed up for up to 28 months or until death. The authors had access to information that could potentially identify individual participants after data collection. Institutional Review Board (IRB) approval for the Indiana Biobank (IRB #1105005445) was obtained, and all participants provided written informed consent in accordance with the Declaration of Helsinki.

### Study outcomes

The primary outcome was mortality. The secondary outcome was AKI status in those who did not have a diagnosis code of end-stage kidney disease (ESKD) at baseline. AKI was defined according to the Kidney Disease: Improving Global Outcomes (KDIGO) guidelines^13^, based on changes in serum creatinine from pre-enrollment baseline to post-COVID-19 diagnosis. Specifically, a diagnosis of AKI was established if there was an increase in serum creatinine greater than or equal to 0.3 mg/dL within 48 hours or an increase in serum creatinine greater than or equal to 1.5 times the last known value (baseline). ESKD status was based on the International Classification of Diseases (ICD-9 and ICD-10) codes listed within the patient’s medical history documentation.

### Laboratory measures

FGF23 and Klotho were measured using biobanked plasma samples as described previously. Total circulating FGF23 levels were measured in ethylenediaminetetraacetic acid (EDTA) plasma using a solid-phase sandwich enzyme-linked immunosorbent assay (ELISA) that detects both C-terminal and intact FGF23 (Cat. No. 60-6100, Quidel, Inc.). FGF23 levels and AKI status were determined concurrently. Klotho was measured in EDTA plasma using an ELISA kit (Cat. No. 27998, Immuno-Biological Laboratories, Inc.). Hematology and inflammation, liver function tests, and blood chemistry information were collected from electronic medical records.

### Statistical analysis

Descriptive statistics were used to describe the baseline characteristics, medications, and laboratory values of the study population. Quantitative variables are presented as mean ± standard deviation or median (interquartile range), and categorical variables are presented as frequencies (%). *P*-values were obtained by ANOVA or the Kruskal-Wallis test for quantitative variables and the chi-square test or Fisher’s exact test for categorical variables. The association between log(FGF23) and age was analyzed via Pearson’s correlation coefficient and simple linear regression. Logistic regression was used to assess the association of FGF23 with AKI status. Cox proportional hazards regression was employed to initially screen significant variables associated with mortality. This was followed by stratified Cox regression to estimate the association between FGF23 and mortality. Kidney status (without AKI, AKI, and ESKD) was specified as the stratification variable in the multivariable Cox model, allowing separate baseline hazard functions across these groups. Adjustment for covariates in baseline mortality hazards in the AKI, without AKI, and ESKD groups was based on univariate regression analysis. *P*-values <0.05 were considered statistically significant. All analyses were performed using SAS v9.4 (SAS Institute).

## RESULTS

### Study population characteristics

Baseline characteristics of the study population are described in Table 1. Of the 111 patients included in this study, 17 (15%) developed AKI, 77 (69%) did not develop AKI, and 17 (15%) had ESKD. Within the AKI group, 53% had a pre-existing CKD diagnosis prior to admission, compared to 31% in the group without AKI (*P* = 0.09). Among the 17 patients with ESKD, 14 (82%) were on dialysis, whereas only one (6%) of the patients who developed AKI was on dialysis. Patients with ESKD had lower BMI (25.7 kg/m^2^; *P* = 0.02) and a higher prevalence of diabetes (88%; *P* = 0.04) and cardiovascular disease (94%; *P* = 0.03) compared to patients with and without AKI. There were no significant differences in age (*P* = 0.85), sex (*P* = 0.58), race (*P* = 0.89), or blood pressure indices (*P* = 0.44) between the three groups. Patients with AKI and ESKD had higher use of calcium channel blockers (53%; *P* = 0.03), and those with ESKD had higher use of α-blockers (18%; *P* = 0.01) compared to patients without AKI. Patients with ESKD had lower use of loop diuretics (29%; *P* = 0.02). There were no significant differences in the use of statins, ACE inhibitors, β-blockers, centrally acting agonists, aldosterone receptor antagonists, potassium-sparing diuretics, or thiazide diuretics between the three groups (*P* > 0.05).

**Table 1:** Baseline characteristics of the study population.

Variable	n	Without AKI	With AKI	ESKD	*P*-value
No. of Subjects		77	17	17	
***Demographics***					
Age, years	111	66 ± 15	67 ± 18	67 ± 13	0.85
Male sex	111	44 (57)	8 (47.1)	11 (65)	0.58
Race					
Black or African American	111	36 (47)	9 (53)	10 (59)	
White		37 (48)	8 (47)	7 (41)	
Other/Unknown		4 (5)	0 (0)	0 (0)	0.89
Hispanic ethnicity	111	5 (7)	0 (0)	1 (6)	0.88
BMI, kg/m^2^	101	32.6 (28; 39)	30.6 (24; 37)	25.7 (23; 32)	0.02*
Systolic BP, mmHg	94	127.5 ± 23.0	117.8 ± 22.0	134.5 ± 27.4	0.15
Diastolic BP, mmHg	94	71.1 ± 18.5	66.3 ± 13.0	67.7 ± 20.0	0.58
MAP, mmHg	94	89.9 ± 18.3	83.5 ± 13.7	90.0 ± 19.0	0.44
HR, bpm	102	84.6 ± 17.0	81.3 ± 16.8	76.6 ± 13.7	0.19
Ever smoker	76	22 (41)	8 (62)	5 (56)	0.36
On dialysis	111	0 (0)	1 (6)	14 (82)	<0.001***
***Comorbidities***					
Hypertension	111	61 (79)	15 (88)	17 (100)	0.10
Diabetes	111	43 (56)	11 (65)	15 (88)	0.04*
Chronic kidney disease	111	24 (31)	9 (53)	17 (100)	<0.001***
Cardiovascular disease	111	52 (68)	15 (88)	16 (94)	0.03*
***Medications***					
Statin	111	39 (51)	11 (65)	11 (65)	0.39
Angiotensin II receptor blocker	111	6 (8)	4 (24)	0 (0)	0.06
ACE inhibitor	111	14 (18)	2 (12)	2 (12)	0.79
α-Blocker	111	1 (1)	1 (6)	3 (18)	0.02*
β-Blocker	111	23 (30)	9 (53)	6 (35)	0.19
Calcium channel blocker	111	21 (27)	9 (53)	9 (53)	0.03*
Central α2-adrenergic agonist	111	4 (5)	2 (12)	1 (6)	0.59
Aldosterone receptor antagonist	111	4 (5)	2 (12)	0 (0)	0.43
Loop diuretic	111	35 (46)	13 (77)	5 (29)	0.02*
Potassium-sparing diuretic	111	4 (5)	1 (6)	0 (0)	1.00
Thiazide diuretic	111	8 (10)	4 (24)	1 (6)	0.32

Abbreviations – No symbol: not significant ( *p* ≥ 0.05); AKI: acute kidney injury; BMI: body mass index; BP: blood pressure; ESKD: end-stage kidney disease; HR: heart rate; MAP: mean arterial pressure.

Notes – Data are presented as mean ± SD, median (interquartile range), or frequencies (%). *P*-values were obtained by ANOVA or the Kruskal-Wallis test for quantitative variables, and the chi-square test or Fisher’s exact test for categorical variables. Asterisks indicate significance levels: p < 0.05 (*),

p < 0.01 (**),

p < 0.001 (***).

**Figure 1:**
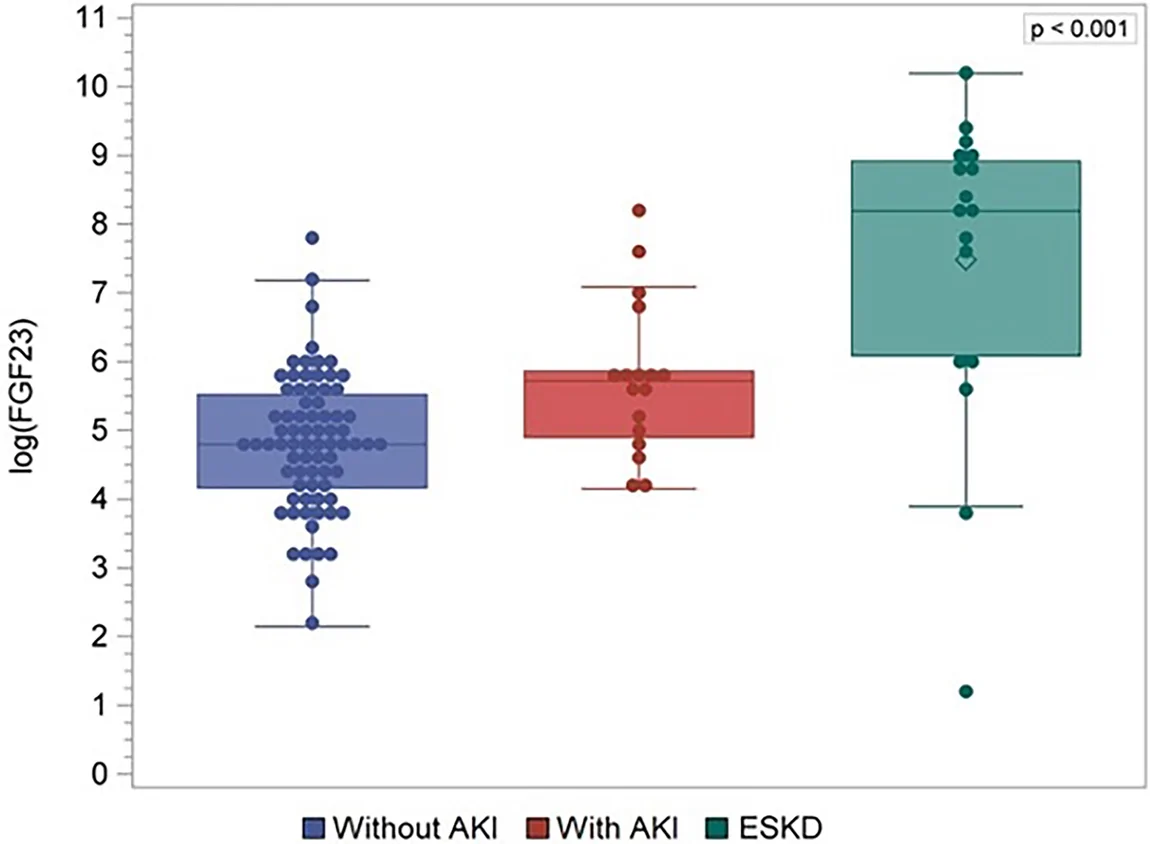
FGF23 levels by AKI status in patients with COVID-19.

Laboratory values are provided in Table 2. Patients with ESKD had lower hematocrit (28.7 ± 5%; *P* = 0.028) and platelet counts (181 [102, 228] K/mm^3^; *P* = 0.02) compared to patients with and without AKI. Creatinine levels (*P* < 0.001) were significantly different among the three groups, with patients with ESKD exhibiting the highest levels (ESKD: 5.1 [2.5, 6.3] mg/dL; AKI: 1.5 [1.2, 1.9] mg/dL; without AKI: 0.9 [0.7, 1.0] mg/dL). Sodium levels were lowest in the ESKD group (134.9 ± 5.3 mmol/L; *P* < 0.002). Procalcitonin and ferritin levels were highest in the ESKD group (1.5 [0.8, 7.3] ng/mL and 1,970 [1,454, 2,425] ng/mL, respectively; *P* < 0.001). C-reactive protein (CRP), erythrocyte sedimentation rate (ESR), interleukin-6, and D-dimer levels were generally higher than the normal range in all groups, which is consistent with the inflammatory state associated with COVID-19^14^. Significantly, FGF23 levels (Table 2 and Figure 1) were elevated in patients with AKI (305.6 [134.6, 350.8] RU/mL) and ESKD (3,607.6 [440.5, 7,452.7] RU/mL) compared with patients without AKI (120.7 [64.3, 249.7] RU/mL; *P* < 0.001). Klotho levels were also significantly different between groups and were lowest in patients with ESKD (with AKI: 594.6 [416; 774] pg/mL; without AKI: 576.1 [455; 730] pg/mL; ESKD: 421.5 [330; 562] pg/mL; *P* = 0.04).

**Table 2:** Laboratory data of the study population.

Variable	n	Without AKI	With AKI	ESKD	*P*-value
***Chemistry***					
Blood urea nitrogen, mg/dL	104	21.0 (14; 33)	40.5 (27; 51)	36.0 (25; 61)	0.003**
Creatinine, mg/dL	111	0.9 (0.7; 1)	1.5 (1; 2)	5.1 (3; 6)	<0.001***
eGFR, ml/min/1.73 m^2^	111	91.0 (69; 101)	40.0 (29; 58)	12.0 (9; 18)	<0.001***
Glucose, mg/dL	102	121.0 (94; 162)	128.0 (97; 186)	111.0 (88; 156)	0.75
Calcium, mg/dL	102	8.4 (8; 9)	8.3 (8; 9)	8.0 (8; 9)	0.43
Sodium, mmol/L	102	139.3 ± 4.0	138.3 ± 5.1	134.9 ± 5.3	0.002**
Potassium, mmol/L	102	4.0 (4; 5)	4.2 (3.8; 4.2)	4.4 (4; 4.5)	0.59
***Liver function***					
Total protein, g/dL	103	6.4 ± 0.8	6.4 ± 1.0	6.1 ± 0.8	0.40
Albumin, g/dL	105	3.0 ± 0.5	2.9 ± 0.7	2.9 ± 0.4	0.73
ALT, U/L	103	29.0 (15; 50)	14.5 (6; 45)	20.0 (11; 26)	0.02*
ALP, U/L	103	72.0 (56; 100)	71.0 (59; 90)	69.0 (51; 111)	0.99
AST, U/L	103	29.0 (22; 56)	25.5 (19; 41)	41.0 (21; 63)	0.34
Bilirubin, mg/dL	103	0.5 (0.5; 0.7)	0.5 (0.4; 0.6)	0.5 (0.4; 0.6)	0.17
***Hematology & Inflammation***					
Hemoglobin, g/dL	104	10.7 ± 1.9	10.2 ± 2.0	9.5 ± 1.4	0.05
Hematocrit, %	104	32.6 ± 5.6	30.6 ± 6.0	28.7 ± 4.6	0.03*
Red blood cells, million/mm^3^	102	3.8 ± 0.7	3.4 ± 0.7	3.2 ± 0.6	0.01*
White blood cells, K/mm^3^	104	8.4 ± 3.8	10.7 ± 5.4	7.7 ± 5.1	0.11
Platelets, K/mm^3^	104	263.0 (164; 355)	209.0 (116; 344)	181.0 (102; 228)	0.02*
CRP, mg/L	93	7.2 (3; 16)	5.4 (2; 12)	10.6 (4; 25)	0.30
ESR, mm/h	79	71.0 (45; 91)	69.0 (45; 96)	51.0 (21; 71)	0.45
PCT, ng/mL	102	0.2 (0.1; 0.5)	0.2 (0.2; 0.7)	1.5 (0.8; 7)	<0.001***
Ferritin, ng/mL	102	445.7 (251; 706)	483.7 (229; 951)	1,970 (1,454; 2,425)	<0.001***
Interleukin-6, pg/mL	53	31.5 (10; 82)	17.0 (5; 85)	22.0 (8; 85)	0.80
D-dimer, ng/mL DDU	104	540.0 (310; 963)	1,727.5 (372; 4,129)	517.0 (326; 912)	0.11
Creatine kinase, U/L	96	127.5 (52; 306)	36.0 (22; 223)	95.5 (55; 350)	0.08
Prothrombin time, sec	59	14.4 (12; 17)	14.0 (13; 15)	13.7 (13; 16)	0.95
Lactate dehydrogenase, U/L	98	340.5 (269; 492)	295.0 (232; 396)	243.0 (200; 543)	0.13
***Biomarkers***					
FGF23, RU/mL	109	120.7 (64; 250)	305.6 (135; 351)	3,607.6 (441; 7,453)	<0.001***
log(FGF23)	109	4.8 ± 1.0	5.7 ± 1.1	7.5 ± 2.3	<0.001***
Klotho, pg/mL	111	576.1 (455; 730)	594.6 (416; 774)	421.5 (330; 562)	0.04*
log(Klotho)	111	6.4 ± 0.4	6.3 ± 0.4	6.0 ± 0.5	0.01*

Abbreviations – AKI: acute kidney injury; ALT: alanine aminotransferase; ALP: alkaline phosphatase; AST: aspartate aminotransferase; CRP: C-reactive protein; DDU: D-dimer units; eGFR: estimated glomerular filtration rate; ESKD: end-stage kidney disease; ESR: erythrocyte sedimentation rate; FGF23: fibroblast growth factor 23; PCT: procalcitonin; RU: relative units.

Notes – Data are presented as mean ± SD, median (interquartile range), or frequencies (%). *P*-values were obtained by ANOVA or the Kruskal-Wallis test for quantitative variables, and the chi-square test or Fisher’s exact test for categorical variables. Asterisks indicate significance levels: *P* < 0.05 (*),

*P* < 0.01 (**),

*P* < 0.001 (***).

No symbol = not significant (*P* ≥ 0.05).

Mean, median, and interquartile ranges of log(FGF23) between patients without AKI, with AKI, and with ESKD. Levels of log(FGF23) were highest in the ESKD group and differed significantly across groups (*P* < 0.001).

### Association of FGF23 with AKI status

Logistic regression analyses evaluating factors associated with AKI status (excluding patients with ESKD) are shown in Table 3. In univariable analysis, higher log(FGF23) levels were significantly associated with greater odds of AKI (OR: 2.30; 95% CI: [1.31, 4.03]; *P* = 0.004). Age, hemoglobin, BMI, diabetes, or cardiovascular disease status were not significantly associated with AKI status (*P* > 0.05) (Table 3 and Supplemental Figure S1).

**Table 3:** Association of FGF23 With AKI status in patients with COVID-19 without end-stage kidney disease.

Variable	n	OR	95% CI	*P*-value
Age, 10-year increase	94	1.07	0.75–1.53	0.70
Age category (ref: <65 years)	94	1.61	0.54–4.79	0.39
Hemoglobin, 1 g/dL increase	87	0.86	0.64–1.15	0.30
Diabetes (ref: No)	94	1.45	0.49–4.32	0.51
Cardiovascular disease (ref: No)	94	3.60	0.77–17.0	0.11
BMI, 1 kg/m^2^ increase	84	0.95	0.89–1.02	0.16
log(FGF23), 1 RU increase	92	2.30	1.31–4.03	0.004**

Abbreviations – BMI: body mass index; FGF23: fibroblast growth factor 23.

Notes – In univariable analysis, patients with high FGF23 levels (excluding patients with ESKD) showed increased odds of having AKI. Asterisks indicate significance levels: *P* < 0.05 (*),

*P* < 0.01 (**),

*P* < 0.001 (***).

No symbol = not significant (*P* ≥ 0.05). Age, hemoglobin, diabetes, cardiovascular disease, and BMI were not associated with AKI status.

### Survival in patients with AKI, without AKI, and with ESKD

**Figure 2:**
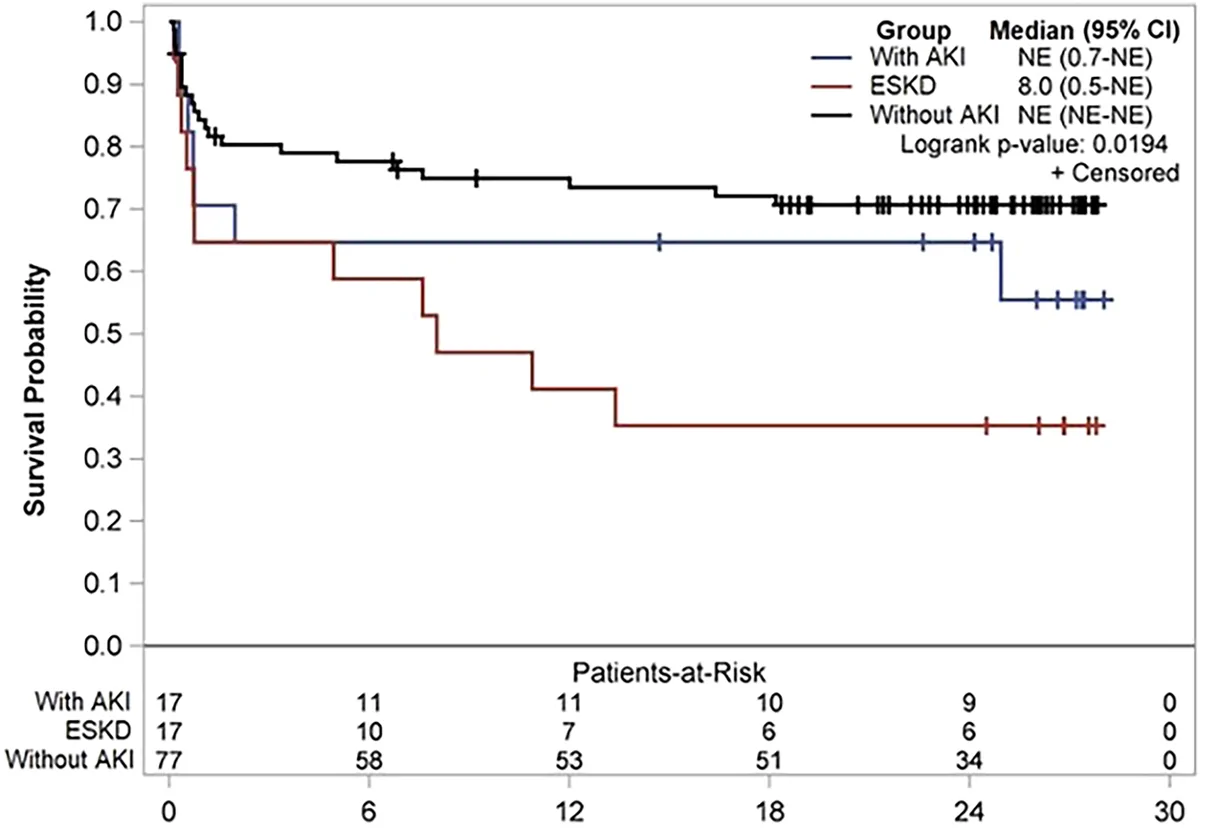
Kaplan-Meier analysis revealed a significant reduction in survival probability in patients with ESKD versus patients without AKI.

Kaplan–Meier analysis demonstrated overall survival in months from the time of enrollment in the biobank to death or last known follow-up (censored) across groups (Figure 2), showing significant differences between groups (log-rank p = 0.019). The median follow-up was 22.6 months. Estimated survival at 1, 3, 6, 12, and 24 months was highest among patients without AKI (84.3%, 80.3%, 77.6%, 74.9%, and 70.6%, respectively), intermediate among those with AKI (70.6%, 64.7%, 64.7%, 64.7%, and 64.7%), and lowest among patients with ESKD (64.7%, 64.7%, 58.8%, 41.2%, and 35.3%).

Overall survival in months from the time of enrollment in the Indiana Biobank to death or the last date known alive (censored patients) between groups. Patients with ESKD had reduced survival compared to patients without AKI (*P* < 0.05). The median follow-up was 22.6 months. Among the 17 patients with ESKD, a total of 11 (65%) died. For the 17 patients with AKI, 7 (41%) died during follow-up. Out of the 77 patients without AKI, 22 (29%) died. NE = not evaluable.

During follow-up, a total of 40 deaths occurred, corresponding to an overall mortality incidence of 36%. Among patients with AKI, 7 of 17 (41%) died. In the ESKD group, 11 of 17 patients (65%) died. Among the 77 patients without AKI, 22 (29%) died. Among patients who died, underlying CKD was present in 4 of 7 (57.1%) with AKI and in 10 of 22 (55.0%) without AKI. Cause-of-death data indicated that deaths were primarily attributed to respiratory disease (26.3%), with 23.7% explicitly related to COVID-19, followed by cardiovascular causes (18.4%) (Supplemental Table S1).

### Association of FGF23 with overall survival

Univariable and stratified Cox regression analyses of FGF23 and overall survival are shown in Table 4. For every ten-year increase in patient age, the unadjusted hazard ratio (HR) for death was 1.88 (95% CI: [1.43, 2.47]; *P* < 0.001). For every unit increase in log(FGF23), the unadjusted HR for death was 1.42 (95% CI: [1.18, 1.71]; *P* < 0.001). Using the group without AKI as the reference group, the unadjusted HR for death was 1.52 (95% CI: [0.65, 3.56]; *P* = 0.30) for patients with AKI and 2.70 (95% CI: [1.31, 5.58]; *P* = 0.007) for patients with ESKD. Cardiovascular disease (HR = 3.40; 95% CI: [1.21, 9.56]; *P* = 0.020) and BMI (HR = 0.79; 95% CI: [0.65, 0.95]; *P* = 0.01) also had significant associations with mortality. In a multivariable model, the significant associations with cardiovascular disease and BMI disappeared (*P* > 0.05). After controlling for patient age, cardiovascular disease, and BMI, and considering different baseline death hazards across the AKI/ESKD groups, log(FGF23) remained significantly associated with an increased risk of death among all patients (HR: 1.45; 95% CI: [1.02, 2.07]; *P* = 0.04). In addition, in an exploratory analysis, no association was found between Klotho and AKI (*P* = 0.41) (Supplemental Figures S2 and S3).

**Table 4:** Association of FGF23 with higher mortality in patients with COVID-19.

Model Type	Variable	*n*	HR	95% CI	*P*-value
Univariable Cox regression	Age, 10-year increase	111	1.88	1.43–2.47	<0.001***
	Age category (ref: <65 years)	111	7.30	2.85–18.7	<0.001***
	Hemoglobin, 1 g/dL increase	104	0.94	0.79–1.12	0.47
	Diabetes (ref: No)	111	1.81	0.90–3.62	0.09
	Cardiovascular disease (ref: No)	111	3.40	1.21–9.56	0.02*
	BMI, 5 kg/m^2^ increase	101	0.79	0.65–0.95	0.01*
	log(FGF23), 1 RU increase	109	1.42	1.18–1.71	<0.001***
	Group AKI (ref: without AKI) ESKD (ref: without AKI)	111	1.52 2.70	0.65–3.56 1.31–5.58	0.03*
Multivariable stratified Cox regression	Age, 10-year increase	99	1.81	1.31–2.51	<0.001***
	Cardiovascular disease (ref: No)		0.95	0.31–2.91	0.93
	BMI, 5 kg/m^2^ increase		0.86	0.69–1.09	0.21
	log(FGF23), 1 RU increase		1.45	1.02–2.07	0.04*

Abbreviations – AKI: acute kidney injury; BMI: body mass index; ESKD: end-stage kidney disease; FGF23: fibroblast growth factor 23; RU: relative units.

Notes – After controlling for patient age, history of cardiovascular disease, and BMI, and considering different baseline death hazards across the AKI/ESKD groups, log(FGF23) was significantly associated with an increased risk of death among all patients. Asterisks indicate significance levels: *P* < 0.05 (*),

*P* < 0.01 (**),

*P* < 0.001 (***).

No symbol = not significant (*P* ≥ 0.05).

## DISCUSSION

To the best of our knowledge, this is the first study to demonstrate that elevated circulating FGF23 levels are significantly associated with both AKI status and mortality in patients hospitalized with COVID-19. The association between elevated FGF23 levels and AKI status is particularly noteworthy, given that AKI is highly prevalent in hospitalized patients and can be associated with high mortality and substantial healthcare costs due to prolonged hospitalization^6^. These complications can be exacerbated by delayed AKI diagnosis. Thus, early detection of AKI is critical and can help facilitate early intervention and potentially lead to better outcomes^15^. The potential of a biomarker, such as FGF23, to assess the risk of developing AKI in patients with COVID-19 is critically important, especially given the high mortality rates among those hospitalized or admitted to the ICU^16^. Circulating FGF23 exists in several forms: an intact, biologically active form, which is then proteolytically cleaved into inactive N- and C-terminal fragments^17^. We utilized a C-terminal FGF23 assay capable of capturing both active and inactive forms.

Although the incidence of AKI can vary depending on the criteria utilized and the clinical setting (ICU versus non-ICU), recent studies across Europe and the United States have suggested that the incidence of AKI may reach 20% in hospitalized patients and could exceed 50% in ICU patients with COVID-19^18,19^. In our cohort, we observed a slightly lower incidence of AKI (18%) in patients with COVID-19. Similarly, recent publications have noted significant variability in the reported incidence of AKI among patients admitted with COVID-19. For example, Ng *et al*. observed that among 9,657 patients hospitalized with COVID-19, 40% developed AKI^3^. Discrepancies in the incidence of AKI among patients with COVID-19 can be attributed to various factors, including the geographic location of the study, timing of observations, and disease severity. Notably, studies from Asia tend to report lower AKI rates compared to those conducted in Europe and the United States^18,19^. These differences are likely due to variations in hospitalization criteria and the overall health status of patients upon admission. In our study, we observed a higher prevalence of comorbid conditions, including diabetes mellitus and cardiovascular disease, among patients with AKI. Within the AKI group, 53% had a pre-existing CKD diagnosis prior to admission, compared to 31% in the group without AKI. Similarly, Ng et al.^3^ also demonstrated that patients who developed AKI had a higher prevalence of comorbid conditions such as diabetes mellitus, coronary artery disease, heart failure, and CKD.

FGF23 was initially identified as a key regulator of phosphate metabolism, but subsequent studies have revealed its significant role in various metabolic pathways. Elevated FGF23 levels are commonly observed in patients with CKD, although the mechanisms behind this are not well understood^20^. Elevated FGF23 levels are independently linked to an increased risk of cardiovascular events, progression to ESKD, premature allograft loss after transplantation, and higher mortality among patients with CKD^21,22^. Emerging evidence suggests that FGF23 levels are elevated in patients with AKI, indicating its potential as a prognostic biomarker^6,12^. Our results show that FGF23 levels were significantly higher in patients with COVID-19 with AKI and ESKD compared to those without AKI. Additionally, elevated FGF23 levels were associated with AKI status and higher mortality among all patients hospitalized with COVID-19. These findings underscore the potential of FGF23 as a biomarker associated with AKI status and mortality, which could lead to improved risk assessment and management strategies. In contrast, Klotho levels were significantly lower in patients with ESKD, indicating a potential link to the severity of kidney impairment. Although Klotho has been considered a potential early biomarker and therapeutic agent for AKI^23^, our study found no association between Klotho levels and AKI status.

Interestingly, studies in animal models and humans have suggested that the increase in FGF23 levels after AKI precedes changes in other classic biomarkers of kidney function, such as neutrophil gelatinase-associated lipocalin (NGAL) in mice and serum creatinine and other mineral metabolites in patients who developed AKI following cardiac surgery or critical illness^6,12^. Further studies are needed to better understand how FGF23 levels compare to other biomarkers in AKI, such as KIM-1. Importantly, FGF23 has also emerged as a key regulator with bidirectional relationships involving anemia, iron status, and inflammation, indicating its potential role in the pathophysiology of AKI^24,25^. Elevated FGF23 levels in AKI may contribute to the inflammatory environment typical of AKI. Increased FGF23 levels, as an inflammatory mediator, can worsen kidney damage by promoting vascular calcification and intensifying inflammation^26^. This creates a feedback loop where inflammation exacerbates AKI, raising FGF23 levels and perpetuating injury^24^. Recent research highlights the adverse effects of FGF23 on iron metabolism^25,27^. Elevated FGF23 stimulates hepcidin production, reducing intestinal iron absorption and blood iron levels. Conversely, iron deficiency or anemia may increase FGF23, creating a harmful feedback loop. These interactions can impede kidney recovery from acute injury, negatively affecting erythropoiesis and metabolic health^25^. Additional mechanisms of concern involve the role of elevated FGF23 levels in inducing profibrotic signals in injury-stimulated fibroblasts at the kidney, potentially contributing to the progression of kidney injury beyond the initial insult. Furthermore, FGF23 has also been implicated in increased myofibroblast activity during AKI, possibly promoting the signaling cascade that leads to renal fibrosis. Higher plasma FGF23 levels are also associated with an increased risk of infection, possibly through modulation of the innate immune response. Therefore, FGF23 may be involved in the progression of AKI in the context of COVID-19 infection, although this hypothesis was not directly examined in the current study. Collectively, these factors underscore the complex adverse effects of elevated FGF23 on kidney health.

Our cohort exhibited a mortality rate of 41% among patients with AKI, which aligns with similar findings reported in other studies. Organ dysfunction, particularly involving the lungs and kidneys, is a key indicator of COVID-19 severity, and it is associated with significantly elevated mortality rates^28^. AKI is regarded as a negative prognostic indicator for survival, especially among patients in the ICU^29^. Additionally, patients with ESKD experience more severe symptoms and have an increased risk of mortality from COVID-19^30^. Consistent with the literature, we observed a higher mortality rate of 65% in patients with ESKD within our cohort. However, across Europe, patients with ESKD and COVID-19 have reported mortality rates ranging from 20% to 30%^27^. Similarly, the variability observed in incidence rates is mirrored in discussions surrounding mortality. This divergence can be attributed to several factors, including prolonged hospitalization, a large and racially or ethnically diverse sample population, and differences in access to healthcare and resource allocation. These alarming results highlight the urgent need for strategies to assess the risk of AKI associated with COVID-19 infection. Early detection of AKI, combined with appropriate therapeutic measures, is crucial for mitigating adverse outcomes and reducing the high mortality rate among patients with COVID-19.

The results of our study should be interpreted against its strengths and limitations. The present study has several notable strengths. It is the first prospective, longitudinal study to assess FGF23 levels in patients with COVID-19 in relation to clinically important outcomes, including AKI and mortality. Additional strengths include a well-characterized cohort and a study design that encompasses three arms: patients without AKI, patients with AKI, and individuals with ESKD. This approach allows for a comprehensive assessment of FGF23’s role across varying degrees of kidney impairment, enhancing the robustness of our findings. The study has several limitations. One significant limitation is the small cohort size, which was constrained by the availability of patients in the Indiana Biobank. Additionally, FGF23 concentrations were measured exclusively at baseline due to the absence of serial specimens in the biobank, thereby limiting the evaluation of longitudinal changes and their relationship with AKI progression. Furthermore, corticosteroid use and mechanical ventilation—which were prevalent in COVID-19 management during 2020—were not consistently captured in the biobank dataset and therefore could not be included in the multivariable analyses.

Finally, the study lacked access to other intermediate endpoints, such as echocardiographic data, which could provide valuable insights into meaningful outcomes related to kidney health and cardiovascular function.

In conclusion, our findings demonstrate that elevated circulating FGF23 levels are significantly associated with AKI status and higher mortality in patients hospitalized with COVID-19. Specifically, FGF23 levels were markedly higher in patients with AKI and ESKD compared to those without AKI. These results highlight the potential of FGF23 as a critical biomarker for assessing the risk of AKI and mortality in patients with COVID-19, paving the way for improved risk assessment and management strategies. Furthermore, additional research is essential to gain a comprehensive understanding of FGF23’s role in guiding clinical decision-making and enhancing outcomes for patients at risk of AKI, including its potential association with subsequent CKD progression, as well as the mechanisms underlying the pathophysiology of AKI.

## Data availability

The datasets generated and/or analyzed during the current study are not publicly available due to privacy restrictions but are available from the corresponding author upon reasonable request.

## SUPPLEMENTARY MATERIAL

The following online material is available for this article:

- Table S1 - Distribution of primary causes of death (n = 40).
- Figure S1 - Correlation between FGF23 and age in patients with COVID-19.
- Figure S2 - Klotho levels by AKI status in patients with COVID-19.
- Figure S3 - Kaplan-Meier analysis showed no significant difference in survival probability between patients with COVID-19 grouped by Klotho and AKI status.
