# Supplementary material for: Elevated circulating fibroblast growth factor 23 is associated with acute kidney injury and mortality in coronavirus disease 2019 (COVID-19)
Source: J Bras Nefrol. 2026 Aug 24;48(4):e20250377. doi: 10.1590/2175-8239-JBN-2025-0377en (PMC13506166; doi:10.1590/2175-8239-JBN-2025-0377en)
Supplement: Supplementary file 1 [file 2175-8239-jbn-48-4-e20250377-pt-suppl1.pdf]

Braz. J. Nephrol.

https://doi.org/10.1590/2175-8239-JBN-2025-0377pt

Material Suplementar para "Níveis elevados de fator de crescimento de fibroblastos 23 e sua associação com lesão renal aguda e mortalidade na doença por coronavírus 2019 (COVID-19)"

Tabela S1 - Distribuição das principais causas de óbito (n = 40).

	Frequência	Percentual (%)
Relacionada à COVID-19	9	23,7
Doença cardiovascular	7	18,4
Doença respiratória	10	26,3
Doença renal	3	7,9
Sepse/infecções	3	7,9
Outras causas	6	15,8

Dados faltantes: n = 2
