# Supplementary material for: Elevated circulating fibroblast growth factor 23 is associated with acute kidney injury and mortality in coronavirus disease 2019 (COVID-19)
Source: J Bras Nefrol. 2026 Aug 24;48(4):e20250377. doi: 10.1590/2175-8239-JBN-2025-0377en (PMC13506166; doi:10.1590/2175-8239-JBN-2025-0377en)
Supplement: Supplementary file 2 [file 2175-8239-jbn-48-4-e20250377-pt-suppl2.pdf]

Braz. J. Nephrol.
https://doi.org/10.1590/2175-8239-JBN-2025-0377pt

Material Suplementar para “Níveis elevados de fator de crescimento de fibroblastos 23 e sua associação com lesão renal aguda e mortalidade na doença por coronavírus 2019 (COVID-19)”

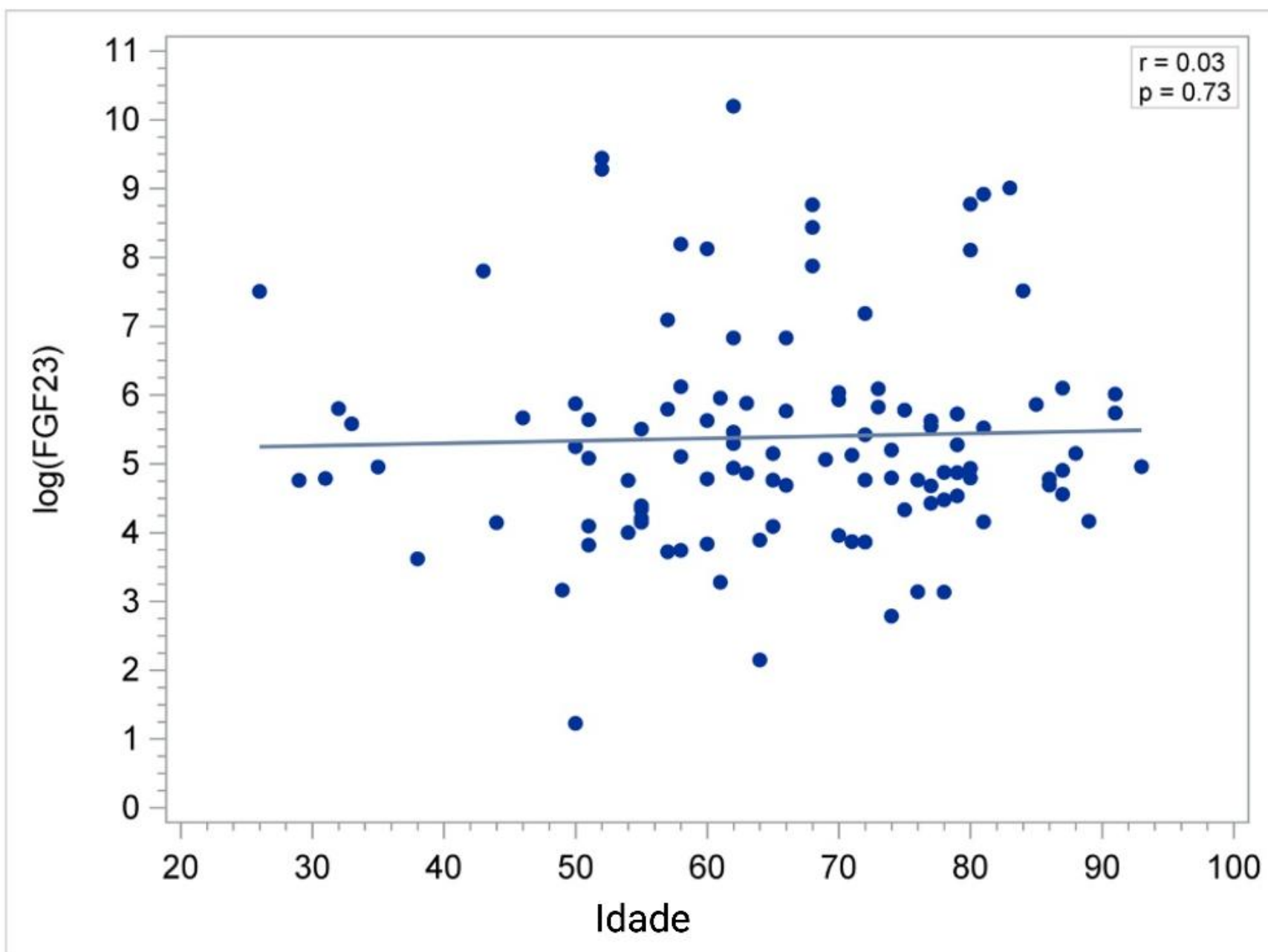

Figura S1 - Correlação entre FGF23 e idade em pacientes com COVID-19.

Correlação linear entre log(FGF23) e idade em toda a coorte de pacientes com COVID-19. log(FGF23) não se associou à idade (r = 0,03; p = 0,73).
