# Supplementary material for: Elevated circulating fibroblast growth factor 23 is associated with acute kidney injury and mortality in coronavirus disease 2019 (COVID-19)
Source: J Bras Nefrol. 2026 Aug 24;48(4):e20250377. doi: 10.1590/2175-8239-JBN-2025-0377en (PMC13506166; doi:10.1590/2175-8239-JBN-2025-0377en)
Supplement: Supplementary file 5 [file 2175-8239-jbn-48-4-e20250377-suppl1.pdf]

Braz. J. Nephrol.
https://doi.org/10.1590/2175-8239-JBN-2025-0377en

Supplementary Material to "Elevated circulating fibroblast growth factor 23 is associated with acute kidney injury and mortality in coronavirus disease 2019 (COVID-19)"

Table S1 - Distribution of Primary Causes of Death (n = 40).

	Frequency	Percent (%)
Covid-19 Related	9	23.7
Cardiovascular disease	7	18.4
Respiratory disease	10	26.3
Renal disease	3	7.9
Sepsis/Infections	3	7.9
Other causes	6	15.8

Frequency Missing = 2.
