# Supplementary material for: Elevated circulating fibroblast growth factor 23 is associated with acute kidney injury and mortality in coronavirus disease 2019 (COVID-19)
Source: J Bras Nefrol. 2026 Aug 24;48(4):e20250377. doi: 10.1590/2175-8239-JBN-2025-0377en (PMC13506166; doi:10.1590/2175-8239-JBN-2025-0377en)
Supplement: Supplementary file 6 [file 2175-8239-jbn-48-4-e20250377-suppl2.pdf]

Braz. J. Nephrol.
https://doi.org/10.1590/2175-8239-JBN-2025-0377en

Supplementary Material to "Elevated circulating fibroblast growth factor 23 is associated with acute kidney injury and mortality in coronavirus disease 2019 (COVID-19)"

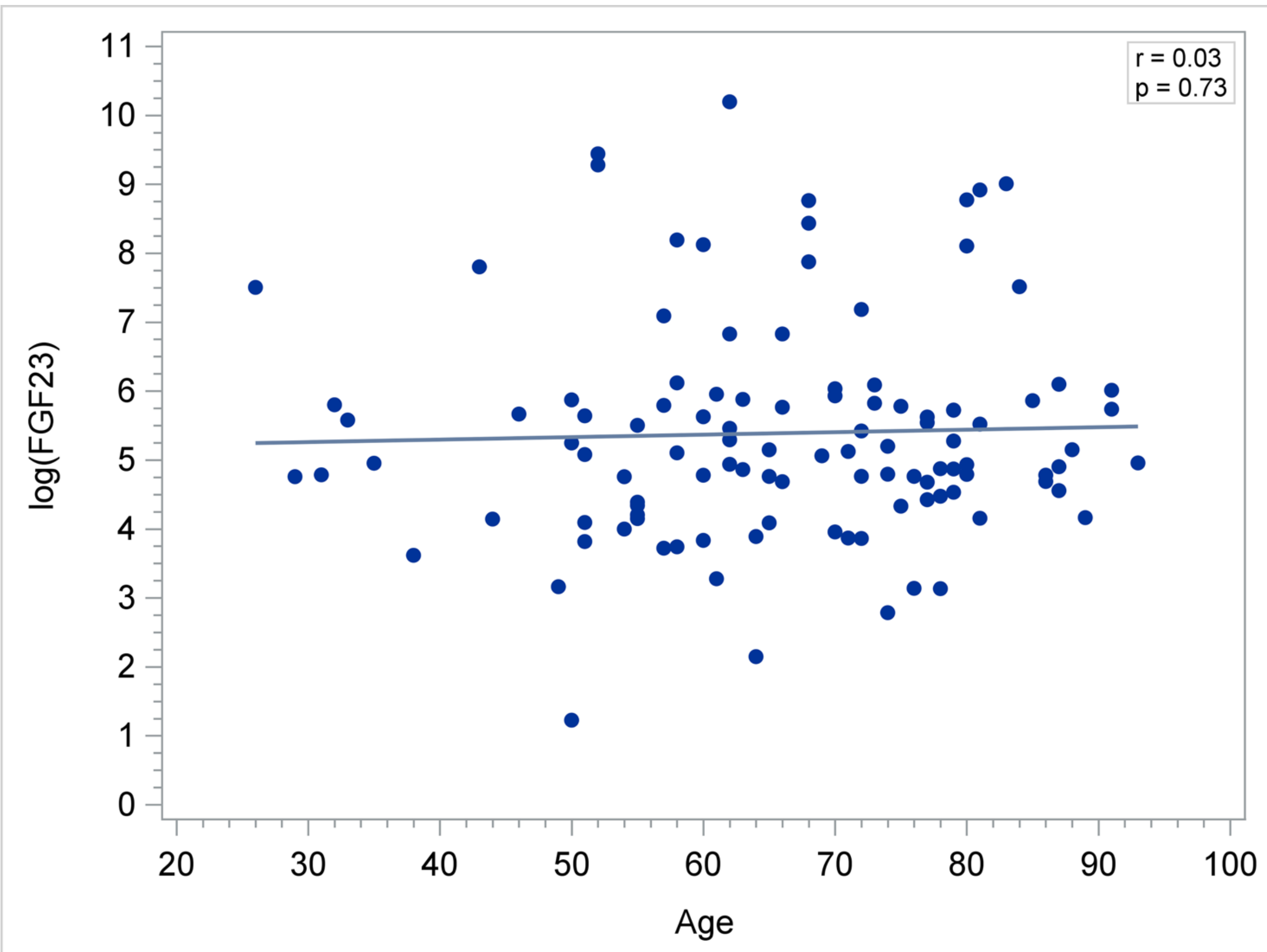

Figure S1 - Correlation between FGF23 and Age in patients with COVID-19.

Linear correlation of log (FGF23) and age in the entire COVID-19 cohort. Log (FGF23) is not associated with age (r=0.03, *P*=0.73).
