# Supplementary material for: Elevated circulating fibroblast growth factor 23 is associated with acute kidney injury and mortality in coronavirus disease 2019 (COVID-19)
Source: J Bras Nefrol. 2026 Aug 24;48(4):e20250377. doi: 10.1590/2175-8239-JBN-2025-0377en (PMC13506166; doi:10.1590/2175-8239-JBN-2025-0377en)
Supplement: Supplementary file 7 [file 2175-8239-jbn-48-4-e20250377-suppl3.pdf]

Braz. J. Nephrol.
https://doi.org/10.1590/2175-8239-JBN-2025-0377en

Supplementary Material to "Elevated circulating fibroblast growth factor 23 is associated with acute kidney injury and mortality in coronavirus disease 2019 (COVID-19)"

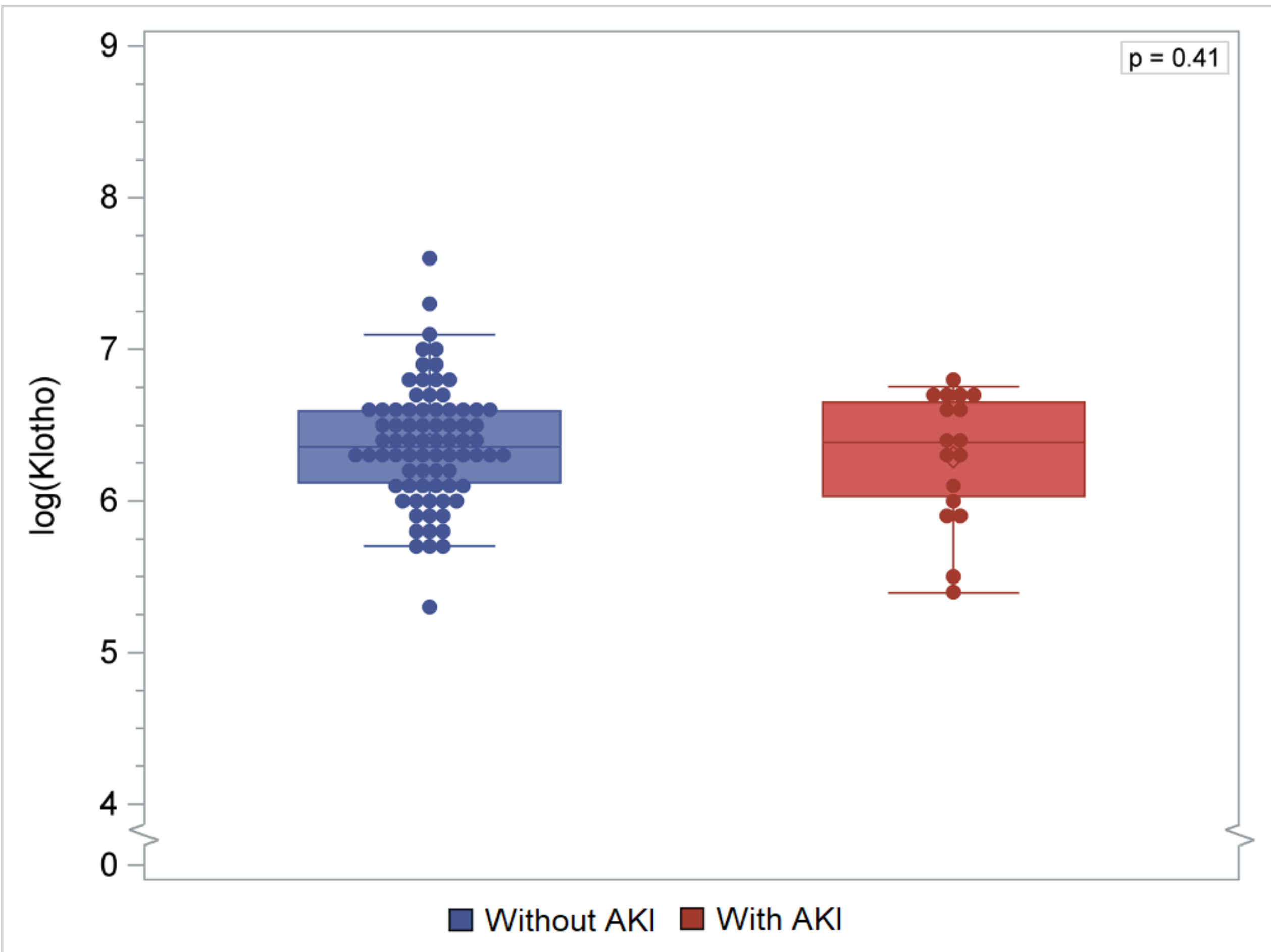

Figure S2 - Klotho levels by AKI status in patients with COVID-19.

Mean, median, and interquartile ranges of log (Klotho) between patients without AKI and with AKI. Log (Klotho) is not significantly different between groups (*P*=0.41).
