# Supplementary material for: Elevated circulating fibroblast growth factor 23 is associated with acute kidney injury and mortality in coronavirus disease 2019 (COVID-19)
Source: J Bras Nefrol. 2026 Aug 24;48(4):e20250377. doi: 10.1590/2175-8239-JBN-2025-0377en (PMC13506166; doi:10.1590/2175-8239-JBN-2025-0377en)
Supplement: Supplementary file 8 [file 2175-8239-jbn-48-4-e20250377-suppl4.pdf]

Braz. J. Nephrol.
https://doi.org/10.1590/2175-8239-JBN-2025-0377en

Supplementary Material to "Elevated circulating fibroblast growth factor 23 is associated with acute kidney injury and mortality in coronavirus disease 2019 (COVID-19)"

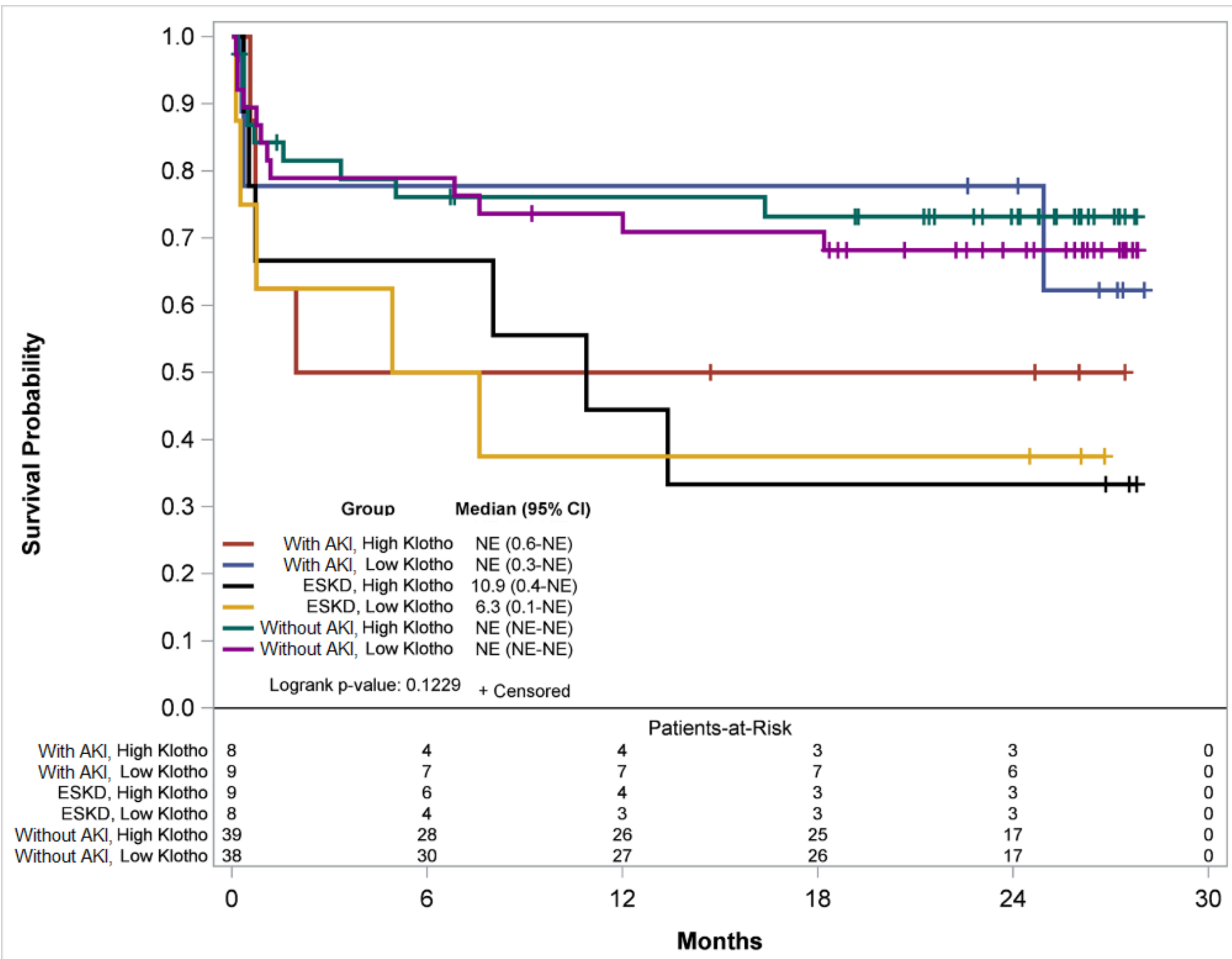

Figure S3 - Kaplan-Meier analysis showed no significant difference in survival probability between patients with COVID-19 grouped by Klotho and AKI status.

Overall survival in months from time of enrollment in the Indiana biobank to death or last date known alive (censored patients) between groups. NE = Not Evaluable.
