# Supplementary material for: Elevated circulating fibroblast growth factor 23 is associated with acute kidney injury and mortality in coronavirus disease 2019 (COVID-19)
Source: J Bras Nefrol. 2026 Aug 24;48(4):e20250377. doi: 10.1590/2175-8239-JBN-2025-0377en (PMC13506166; doi:10.1590/2175-8239-JBN-2025-0377en)
Supplement: Translation

# Translation: Níveis elevados de fator de crescimento de fibroblastos 23 e sua associação com lesão renal aguda e mortalidade na doença por coronavírus 2019 (COVID-19)

**DOI:** 10.1590/2175-8239-JBN-2025-0377pt

**Authors:** Gayatri Narayanan, Monique Opuszcka Campos, Nolan Groninger, Heather N. Burney, Matthew Hays, Matthew Dollins, Eliott Arroyo, Arvin Halim, Yang Li, Syed Jawad Sher, Sharon Moe, Kenneth Lim

**Affiliations:** 1 Indiana University School of Medicine, Department of Medicine, Division of Nephrology & Hypertension, Indianapolis, Estados Unidos.; 2 Indiana University School of Medicine, Department of Biostatistics and Health Data Science, Indianapolis, Estados Unidos.; 3 Wake Forest University, Department of Health and Exercise Science, Winston-Salem, Estados Unidos.

**Keywords:** Injúria Renal Aguda, Biomarcadores, COVID-19, Fator de Crescimento de Fibroblastos 23, Mortalidade

## Abstract

**Introdução:**

Lesão renal aguda (LRA) é um importante fator prognóstico negativo para a sobrevida em pacientes hospitalizados com COVID-19, porém existem poucos biomarcadores confiáveis para LRA e mortalidade. Estudo teve como objetivo avaliar se níveis elevados de fator de crescimento de fibroblastos 23 (FGF23) estão associados à ocorrência de LRA e à mortalidade em pacientes com COVID-19.

**Métodos:**

Nesta coorte prospectiva de 111 pacientes com COVID-19 hospitalizados no *Indiana University Academic Health Center* (abril–outubro de 2020), os níveis circulantes de FGF23 e de seu correceptor Klotho foram avaliados quanto à sua associação com LRA e com a sobrevida em 28 meses.

**Resultados:**

Dos 111 pacientes, 77 não apresentaram LRA (91,0 [69,2–101,3] mL/min/1,73 m^2^), 17 apresentaram LRA (39,7 [28,7–57,8] mL/min/1,73 m^2^) e 17 apresentavam doença renal crônica terminal (DRT; 11,9 [8,6–17,5] mL/min/1,73 m^2^). O tempo mediano de seguimento foi de 22,6 meses. Os níveis medianos de FGF23 foram mais elevados em pacientes com LRA (305,6 [134,6–350,8] RU/mL) e em pacientes com DRT (3.607,6 [440,5–7.452,7] RU/mL), em comparação com aqueles sem LRA (120,7 [64,3–249,7] RU/mL; P < 0,001). Pacientes (excluindo aqueles com DRT) com níveis elevados de FGF23 apresentaram maior probabilidade de LRA (OR: 2,30; IC 95%: [1,31–4,03]; P = 0,004). Além disso, cada aumento de uma unidade no log(FGF23) esteve associado a um risco 45% maior de mortalidade na coorte (HR: 1,45; IC 95%: [1,02–2,07]; P = 0,04), após ajuste para idade, doença cardiovascular e IMC. Os níveis de Klotho diferiram entre os grupos (sem LRA: 576,1 [455, 730] pg/mL; com LRA: 594,6 [416,774] pg/mL; DRT: 421,5 [330, 562] pg/mL; P = 0,04), mas não estiveram associados à presença de LRA.

**Conclusão:**

Níveis elevados de FGF23 estão associados à presença de LRA e à mortalidade em pacientes com COVID-19.

## INTRODUÇÃO

Em 2019, o surgimento de um agente infeccioso conhecido como vírus SARS-CoV-2, responsável pela doença por coronavírus 2019 (COVID-19), levou a uma pandemia global que já acumulou mais de 600 milhões de casos e resultou em mais de 6,5 milhões de óbitos em todo o mundo^1^. Desde então, a COVID-19 permaneceu como a terceira principal causa de morte entre 2020 e 2022^2^. A doença apresenta natureza biologicamente versátil, podendo afetar diretamente diversos sistemas orgânicos, incluindo os sistemas respiratório, cardiovascular e urinário. Uma das principais complicações associadas à COVID-19 é a lesão renal aguda (LRA). A LRA desenvolve-se como complicação secundária em aproximadamente 20–40% dos pacientes com COVID-19 em estado crítico, e até 20% dos pacientes com COVID-19 admitidos em unidade de terapia intensiva (UTI) necessitam de terapia de substituição renal (TSR)^3^. Além disso, pacientes com COVID-19 que desenvolvem LRA apresentam risco significativamente maior de mortalidade intra-hospitalar em comparação com aqueles sem LRA^3^. Dessa forma, a presença de LRA é considerada um importante fator prognóstico negativo para a sobrevida em pacientes internados em UTI. No entanto, até o momento, não existem biomarcadores precoces estabelecidos para avaliar o risco de desenvolvimento de LRA secundária à infecção por COVID-19.

O fator de crescimento de fibroblastos 23 (FGF23) é uma fosfatonina derivada do osso que se eleva nos estágios iniciais da disfunção renal e está intimamente associado à LRA e à mortalidade em pacientes em estado crítico. Níveis elevados de FGF23 atuam contrabalançando o aumento das concentrações de fosfato decorrente da disfunção renal por meio da ligação ao complexo formado pelo correceptor Klotho e pelo receptor 1 do fator de crescimento de fibroblastos (FGFR1) nos rins^4^. Essa interação resulta na supressão dos cotransportadores de sódio-fosfato NaPi2a e NaPi2c nos túbulos proximais, na supressão do cotransportador NaPi2b no intestino, na redução da expressão da 1α-hidroxilase e no aumento da expressão da 24-hidroxilase^5^. Os efeitos resultantes da ação do FGF23 incluem a redução dos níveis de 1,25-di-hidroxivitamina D (calcitriol), a inibição da absorção intestinal de fosfato e o aumento da excreção renal de fosfato, contribuindo para contrabalançar a hiperfosfatemia. De forma relevante, níveis elevados de FGF23 estão independentemente associados ao maior risco de eventos cardiovasculares, e evidências emergentes sugerem que esses níveis elevados também predizem mortalidade intra-hospitalar em pacientes com LRA e doença renal crônica (DRC)^6,7,8,9,10^.

Estudos anteriores investigaram biomarcadores inflamatórios e outros biomarcadores estabelecidos de lesão renal na COVID-19^11^. No entanto, até onde sabemos, a associação entre níveis circulantes de FGF23, presença de LRA e desfechos de mortalidade nessa população ainda não foi explorada. Essa questão torna-se particularmente relevante diante de evidências emergentes de que o FGF23 pode atuar como um novo biomarcador de LRA. Estudos recentes indicam que níveis elevados de FGF23 após LRA podem surgir antes de alterações em biomarcadores clássicos de função renal, como o NGAL em modelos experimentais em camundongos, bem como a creatinina sérica e os metabólitos minerais em pacientes após cirurgia cardíaca e naqueles em estado crítico^6,12^. No entanto, ainda não está claro se esse fenômeno biológico também está presente e associado a desfechos clínicos no contexto da COVID-19. Assim, estudos que contribuam para aprimorar a avaliação do risco de desenvolvimento de LRA e para uma melhor estimativa do risco de mortalidade em pacientes com COVID-19 podem favorecer intervenções precoces, melhorar as estratégias de manejo da LRA e, consequentemente, os desfechos clínicos. Essa questão é particularmente relevante, uma vez que a COVID-19 tornou-se endêmica e permanece como uma importante causa de mortalidade em populações vulneráveis. Nesse contexto, este estudo de coorte prospectivo teve como objetivo avaliar se os níveis de FGF23 estão associados à presença de LRA e à mortalidade em adultos hospitalizados com COVID-19.

## MÉTODOS

### Desenho do estudo e coorte

Trata-se de um estudo de coorte prospectivo envolvendo 111 pacientes com diagnóstico confirmado de COVID-19 hospitalizados no *Indiana University Health Academic Health Center* (Indianapolis, IN, EUA) entre abril e outubro de 2020. Foram incluídos pacientes hospitalizados com diagnóstico confirmado de COVID-19 e idade ≥18 anos. Amostras de sangue foram coletadas no período de até 27 dias após a admissão hospitalar inicial e posteriormente armazenadas no *Indiana Biobank* (Indianapolis, IN, EUA). As amostras e os dados clínicos foram acessados para fins de pesquisa a partir de 4 de abril de 2021. Dados clínicos anonimizados foram fornecidos pelo *Regenstrief Institute*, Inc., organização de informática em saúde que disponibiliza dados provenientes da *Indiana Network for Patient Care* (INPC). Os participantes que atenderam aos critérios de inclusão foram acompanhados por até 28 meses ou até o óbito. Após a coleta dos dados, os autores tiveram acesso a informações que poderiam permitir a identificação individual dos participantes. O estudo foi aprovado pelo Comitê de Ética em Pesquisa (*Institutional Review Board* – IRB) do *Indiana Biobank* (IRB nº 1105005445), e todos os participantes forneceram consentimento informado por escrito, em conformidade com a Declaração de Helsinque.

### Desfechos do estudo

O desfecho primário foi a mortalidade. O desfecho secundário foi a presença de LRA entre os pacientes que não apresentavam diagnóstico prévio de doença renal crônica terminal (DRT) no momento da internação hospitalar. A LRA foi definida de acordo com as diretrizes do *Kidney Disease: Improving Global Outcomes (KDIGO)* ^13^, com base nas alterações da creatinina sérica entre o período pré-inclusão e após o diagnóstico de COVID-19. Especificamente, considerou-se LRA quando houve aumento da creatinina sérica ≥0,3 mg/dL em até 48 horas ou aumento da creatinina sérica ≥1,5 vez em relação ao valor basal previamente conhecido. A presença de DRT foi definida com base nos códigos da *International Classification of Diseases* (ICD-9 e ICD-10) registrados no prontuário médico dos pacientes.

### Análises laboratoriais

Os níveis de FGF23 e Klotho foram mensurados utilizando amostras de plasma armazenadas em biobanco, conforme descrito anteriormente. Os níveis circulantes totais de FGF23 foram determinados em plasma coletado em ácido etilenodiaminotetracético (EDTA) por meio de um ensaio imunoenzimático do tipo *sandwich* em fase sólida (*enzyme-linked immunosorbent assay* – ELISA), capaz de detectar tanto FGF23 C-terminal quanto FGF23 intacto (Cat. nº 60-6100, Quidel, Inc.). Os níveis de FGF23 e a ocorrência de LRA foram avaliados concomitantemente. Os níveis de Klotho foram mensurados em plasma com EDTA utilizando um ensaio ELISA (Cat. nº 27998, *Immuno-Biological Laboratories*, Inc.). Informações referentes a parâmetros hematológicos e inflamatórios, testes de função hepática e análises bioquímicas sanguíneas foram obtidas dos prontuários médicos eletrônicos.

### Análise estatística

Estatísticas descritivas foram utilizadas para descrever as características basais, o uso de medicamentos e os parâmetros laboratoriais da população do estudo. As variáveis quantitativas são apresentadas como média ± desvio padrão ou mediana (intervalo interquartil), enquanto as variáveis categóricas são apresentadas como frequências (%). As comparações entre grupos foram realizadas utilizando ANOVA ou teste de *Kruskal–Wallis* para variáveis quantitativas e os testes do qui-quadrado ou exato de *Fisher* para variáveis categóricas. A associação entre log(FGF23) e idade foi avaliada por meio do coeficiente de correlação de *Pearson* e de regressão linear simples. A regressão logística foi utilizada para avaliar a associação entre FGF23 e a ocorrência de LRA. Modelos de regressão de *Cox* com riscos proporcionais foram inicialmente empregados para identificar variáveis associadas à mortalidade. Posteriormente, utilizou-se regressão de *Cox* estratificada para estimar a associação entre FGF23 e mortalidade. A condição renal dos pacientes (sem LRA, com LRA e com DRT) foi especificada como variável de estratificação no modelo de *Cox* multivariável, permitindo funções de risco basal distintas entre esses grupos. O ajuste para covariáveis nos modelos de mortalidade nos grupos sem LRA, com LRA e com DRT foi baseado em análises de regressão univariada. Valores de p < 0,05 foram considerados estatisticamente significativos. Todas as análises foram realizadas utilizando o *software* SAS versão 9.4 (*SAS Institute*).

## RESULTADOS

### Características da população do estudo

As características basais da população do estudo estão apresentadas na Tabela 1. Dos 111 pacientes incluídos nesta análise, 17 (15%) desenvolveram LRA, 77 (69%) não desenvolveram LRA e 17 (15%) apresentavam DRT. No grupo com LRA, 53% dos pacientes tinham diagnóstico prévio de DRC antes da admissão hospitalar, em comparação com 31% no grupo sem LRA (p = 0,09). Entre os 17 pacientes com DRT, 14 (82%) estavam em diálise, enquanto apenas 1 (6%) dos pacientes que apresentaram LRA estava em diálise. Pacientes com DRT apresentaram menor índice de massa corporal (IMC) (25,7 kg/m^2^; p = 0,02) e maior prevalência de diabetes (88%; p = 0,04) e doença cardiovascular (94%; p = 0,03) em comparação com pacientes com e sem LRA. Não foram observadas diferenças significativas entre os três grupos em relação à idade (p = 0,85), sexo (p = 0,58), raça (p = 0,89) ou pressão arterial (p = 0,44). Pacientes com LRA e DRT apresentaram maior uso de bloqueadores dos canais de cálcio (53%; p = 0,03), e aqueles com DRT apresentaram maior uso de bloqueadores α (18%; p = 0,01) em comparação com pacientes sem LRA. Pacientes com DRT apresentaram menor uso de diuréticos de alça (29%; p = 0,02). Não foram observadas diferenças significativas no uso de estatinas, inibidores da enzima conversora de angiotensina (IECA), β-bloqueadores, agonistas de ação central, antagonistas do receptor de aldosterona, diuréticos poupadores de potássio ou diuréticos tiazídicos entre os três grupos (p > 0,05).

**Tabela 1:** Características basais da população do estudo.

Variável	n	Sem LRA	LRA	DRT	Valor de p
No. de participantes		77	17	17	
***Características demográficas***					
Idade, anos	111	66 ± 15	67 ± 18	67 ± 13	0,85
Sexo masculino	111	44 (57)	8 (47,1)	11 (65)	0,58
Raça					
Negra ou Afro-americana	111	36 (47)	9 (53)	10 (59)	0,89
Branca		37 (48)	8 (47)	7 (41)	
Outras		4 (5)	0 (0)	0 (0)	
Etnia hispânica	111	5 (7)	0 (0)	1 (6)	0,88
IMC, kg/m^2^	101	32,6 (28; 39)	30,6 (24; 37)	25,7 (23; 32)	0,02*
PA sistólica, mmHg	94	127,5 ± 23,0	117,8 ± 22,0	134,5 ± 27,4	0,15
PA diastólica, mmHg	94	71,1 ± 18,5	66,3 ± 13,0	67,7 ± 20,0	0,58
PAM, mmHg	94	89,9 ± 18,3	83,5 ± 13,7	90,0 ± 19,0	0,44
FC, bpm	102	84,6 ± 17,0	81,3 ± 16,8	76,6 ± 13,7	0,19
Tabagismo prévio	76	22 (41)	8 (62)	5 (56)	0,36
Em diálise	111	0 (0)	1 (6)	14 (82)	<0,001***
***Comorbidades***					
Hipertensão	111	61 (79)	15 (88)	17 (100)	0,10
Diabetes	111	43 (56)	11 (65)	15 (88)	0,04*
Doença renal crônica	111	24 (31)	9 (53)	17 (100)	<0,001***
Doença cardiovascular	111	52 (68)	15 (88)	16 (94)	0,03*
***Medicações***					
Estatina	111	39 (51)	11 (65)	11 (65)	0,39
Bloqueador do receptor de angiotensina II	111	6 (8)	4 (24)	0 (0)	0,06
Inibidor da ECA	111	14 (18)	2 (12)	2 (12)	0,79
Bloqueador α	111	1 (1)	1 (6)	3 (18)	0,02*
Bloqueador β	111	23 (30)	9 (53)	6 (35)	0,19
Bloqueador de canal de cálcio	111	21 (27)	9 (53)	9 (53)	0,03*
Agonista α2-adrenérgico central	111	4 (5)	2 (12)	1 (6)	0,59
Antagonista do receptor de aldosterona	111	4 (5)	2 (12)	0 (0)	0,43
Diurético de alça	111	35 (46)	13 (77)	5 (29)	0,02*
Diurético poupador de potássio	111	4 (5)	1 (6)	0 (0)	1,00
Diurético tiazídico	111	8 (10)	4 (24)	1 (6)	0,32

Abreviaturas – LRA: lesão renal aguda; IMC: índice de massa corporal; PA: pressão arterial; DRC: doença renal crônica; FC: frequência cardíaca; PAM: pressão arterial média.

Notas – Os dados são apresentados como média ± desvio padrão, mediana (intervalo interquartil) ou frequências (%). Os valores de p foram obtidos por ANOVA ou teste de *Kruskal-Wallis* para variáveis quantitativas, e teste do qui-quadrado ou teste exato de *Fisher* para variáveis categóricas. Os asteriscos indicam níveis de significância: p < 0,05 (*),

p < 0,01 (**),

p < 0,001 (***).

Ausência de símbolo indica resultado não significativo (p ≥ 0,05).

Os valores laboratoriais estão apresentados na Tabela 2. Pacientes com DRT apresentaram menores níveis de hematócrito (28,7 ± 5%; p = 0,028) e contagem de plaquetas (181 [102, 228] K/mm^3^; p = 0,02) em comparação com pacientes com e sem LRA. Os níveis de creatinina (p < 0,001) diferiram significativamente entre os três grupos, sendo mais elevados nos pacientes com DRT (DRT: 5,1 [2,5, 6,3] mg/dL; LRA: 1,5 [1,2, 1,9] mg/dL; sem LRA: 0,9 [0,7, 1,0] mg/dL). Os níveis de sódio foram mais baixos no grupo com DRT (134,9 ± 5,3 mmol/L; p < 0,002). As concentrações de procalcitonina e ferritina foram mais elevadas no grupo com DRT (1,5 [0,8, 7,3] ng/mL e 1.970 [1.454, 2.425] ng/mL, respectivamente; p < 0,001). Os valores de proteína C-reativa (PCR), velocidade de hemossedimentação (VHS), interleucina-6 e D-dímero encontravam-se, de modo geral, acima da faixa de normalidade em todos os grupos, o que é consistente com o estado inflamatório associado à COVID-19^14^. De forma semelhante, os níveis de FGF23 (Tabela 2 e Figura 1) estavam significativamente mais elevados em pacientes com LRA (305,6 [134,6, 350,8] RU/mL) e com DRT (3.607,6 [440,5, 7.452,7] RU/mL) em comparação com pacientes sem LRA (120,7 [64,3, 249,7] RU/mL; p < 0,001). Os níveis de Klotho também diferiram significativamente entre os grupos, sendo mais baixos em pacientes com DRT (com LRA: 594,6 [416, 774] pg/mL; sem LRA: 576,1 [455, 730] pg/mL; DRT: 421,5 [330,3, 562] pg/mL; p = 0,04).

**Tabela 2:** Dados laboratoriais da população do estudo.

Variável	n	Sem LRA	LRA	DRT	Valor de p
***Bioquímica***					
Nitrogênio ureico no sangue, mg/dL	104	21,0 (14; 33)	40,5 (27; 51)	36,0 (25; 61)	0,003**
Creatinina, mg/dL	111	0,9 (0,7; 1)	1,5 (1; 2)	5,1 (3; 6)	<0,001***
TFGe, ml/min/1,73 m^2^	111	91,0 (69; 101)	40,0 (29; 58)	12,0 (9; 18)	<0,001***
Glicose, mg/dL	102	121,0 (94; 162)	128,0 (97; 186)	111,0 (88; 156)	0,75
Cálcio, mg/dL	102	8,4 (8; 9)	8,3 (8; 9)	8,0 (8; 9)	0,43
Sódio, mmol/L	102	139,3 ± 4,0	138,3 ± 5,1	134,9 ± 5,3	0,002**
Potássio, mmol/L	102	4,0 (4; 5)	4,2 (3,8; 4,2)	4,4 (4; 4,5)	0,59
***Função hepática***					
Proteína total, g/dL	103	6,4 ± 0,8	6,4 ± 1,0	6,1 ± 0,8	0,40
Albumina, g/dL	105	3,0 ± 0,5	2,9 ± 0,7	2,9 ± 0,4	0,73
ALT, U/L	103	29,0 (15; 50)	14,5 (6; 45)	20,0 (11; 26)	0,02*
Fosfatase alcalina (FA), U/L	103	72,0 (56; 100)	71,0 (59; 90)	69,0 (51; 111)	0,99
AST, U/L	103	29,0 (22; 56)	25,5 (19; 41)	41,0 (21; 63)	0,34
Bilirrubina total, mg/dL	103	0,5 (0,5; 0,7)	0,5 (0,4; 0,6)	0,5 (0,4; 0,6)	0,17
***Hematologia e Inflamação***					
Hemoglobina, g/dL	104	10,7 ± 1,9	10,2 ± 2,0	9,5 ± 1,4	0,05
Hematócrito, %	104	32,6 ± 5,6	30,6 ± 6,0	28,7 ± 4,6	0,03*
Eritrócitos, milhões/mm^3^	102	3,8 ± 0,7	3,4 ± 0,7	3,2 ± 0,6	0,01*
Leucócitos, mil/mm^3^	104	8,4 ± 3,8	10,7 ± 5,4	7,7 ± 5,1	0,11
Plaquetas, mil/mm^3^	104	263,0 (164; 355)	209,0 (116; 344)	181,0 (102; 228)	0,02*
PCR, mg/L	93	7,2 (3; 16)	5,4 (2; 12)	10,6 (4; 25)	0,30
VHS, mm/h	79	71,0 (45; 91)	69,0 (45; 96)	51,0 (21; 71)	0,45
Procalcitonina, ng/mL	102	0,2 (0,1; 0,5)	0,2 (0,2; 0,7)	1,5 (0,8; 7)	<0,001***
Ferritina, ng/mL	102	445,7 (251; 706)	483,7 (229; 951)	1.970 (1.454; 2.425)	<0,001***
Interleucina-6, pg/mL	53	31,5 (10; 82)	17,0 (5; 85)	22,0 (8; 85)	0,80
D-dímero, ng/mL DDU	104	540,0 (310; 963)	1.727,5 (372; 4.129)	517,0 (326; 912)	0,11
Creatina quinase, U/L	96	127,5 (52; 306)	36,0 (22; 223)	95,5 (55; 350)	0,08
Tempo de protrombina, s	59	14,4 (12; 17)	14,0 (13; 15)	13,7 (13; 16)	0,95
Desidrogenase láctica, U/L	98	340,5 (269; 492)	295,0 (232; 396)	243,0 (200; 543)	0,13
***Biomarcadores***					
FGF23, RU/mL	109	120,7 (64; 250)	305,6 (135; 351)	3.607,6 (441; 7.453)	<0,001***
log(FGF23)	109	4,8 ± 1,0	5,7 ± 1,1	7,5 ± 2,3	<0,001***
Klotho, pg/mL	111	576,1 (455; 730)	594,6 (416; 774)	421,5 (330; 562)	0,04*
log(Klotho)	111	6,4 ± 0,4	6,3 ± 0,4	6,0 ± 0,5	0,01*

Abreviaturas – LRA: lesão renal aguda; ALT: alanina aminotransferase; FA: fosfatase alcalina; AST: aspartato aminotransferase; PCR: proteína C-reativa; DDU: unidades de D-dímero; TFGe: taxa de filtração glomerular estimada; DRC: doença renal crônica; VHS: velocidade de hemossedimentação; FGF23: fator de crescimento de fibroblastos 23; PCT: procalcitonina; RU: unidades relativas.

Notas – Os dados são apresentados como média ± desvio padrão, mediana (intervalo interquartil) ou frequências (%). Os valores de p foram obtidos por ANOVA ou teste de *Kruskal-Wallis* para variáveis quantitativas, e teste do qui-quadrado ou teste exato de *Fisher* para variáveis categóricas. Os asteriscos indicam níveis de significância: p < 0,05 (*),

p < 0,01 (**),

p < 0,001 (***).

Ausência de símbolo indica resultado não significativo (p ≥ 0,05).

**Figura 1:** Níveis de FGF23 de acordo com a presença de LRA em pacientes com COVID-19.

Média, mediana e intervalos interquartis de log(FGF23) entre pacientes sem LRA, com LRA e com doença renal crônica em estágio terminal. Os níveis de log (FGF23) foram mais elevados no grupo com doença renal crônica em estágio terminal e diferiram significativamente entre os grupos (p < 0,001).

### Associação entre FGF23 e LRA

As análises de regressão logística que avaliaram fatores associados à ocorrência de LRA (excluindo pacientes com DRT) estão apresentadas na Tabela 3. Na análise univariada, níveis mais elevados de log(FGF23) estiveram significativamente associados a maiores chances de LRA (OR: 2,30; IC 95%: [1,31–4,03]; p = 0,004). Idade, hemoglobina, IMC, diabetes ou doença cardiovascular não apresentaram associação significativa com a ocorrência de LRA (p > 0,05) (Tabela 3 e Tabela S1).

**Tabela 3:** Associação do FGF23 com a presença de lra em pacientes com COVID-19 sem doença renal crônica em estágio terminal.

Variável	n	OR	IC 95%	Valor de p
Idade (aumento de 10 anos)	94	1,07	0,75–1,53	0,70
Categoria de idade (ref: <65 anos)	94	1,61	0,54–4,79	0,39
Hemoglobina (aumento de 1 g/dL)	87	0,86	0,64–1,15	0,30
Diabetes (ref: não)	94	1,45	0,49–4,32	0,51
Doença cardiovascular (ref: não)	94	3,60	0,77–17,0	0,11
IMC (aumento de 1 kg/m^2^)	84	0,95	0,89–1,02	0,16
log(FGF23) (aumento de 1 RU)	92	2,30	1,31–4,03	0,004**

Abreviaturas – IMC, índice de massa corporal; FGF23, fator de crescimento de fibroblastos 23.

Notas – Em análise univariada, pacientes com níveis elevados de FGF23 (excluindo aqueles com doença renal crônica em estágio terminal) apresentaram maior chance de LRA. Os asteriscos indicam níveis de significância: p < 0,05 (*),

p < 0,01 (**),

p < 0,001 (***).

Ausência de símbolo indica resultado não significativo (p ≥ 0,05). Idade, hemoglobina, diabetes, doença cardiovascular e IMC não apresentaram associação com a presença de LRA.

### Sobrevida em pacientes com LRA, SEM LRA e COM DRT

A análise de Kaplan-Meier demonstrou a sobrevida global, em meses, desde o momento da inclusão no biobanco até o óbito ou a última avaliação de seguimento (censura), entre os diferentes grupos (Figura 2), evidenciando diferenças significativas entre eles (teste *log-rank*; p = 0,019). O tempo mediano de seguimento foi de 22,6 meses. A sobrevida estimada em 1, 3, 6, 12 e 24 meses foi mais elevada entre pacientes sem LRA (84,3%; 80,3%; 77,6%; 74,9% e 70,6%, respectivamente), intermediária entre aqueles com LRA (70,6%; 64,7%; 64,7%; 64,7% e 64,7%) e mais baixa entre pacientes com DRT (64,7%; 64,7%; 58,8%; 41,2% e 35,3%).

**Figura 2:** A análise de Kaplan-Meier demonstrou redução significativa da probabilidade de sobrevida em pacientes com doença renal crônica em estágio terminal em comparação com aqueles sem LRA.

Sobrevida global em meses desde o momento da inclusão no *Indiana Biobank* até o óbito ou a última data conhecida em vida (pacientes censurados), comparada entre os grupos. Pacientes com doença renal crônica em estágio terminal apresentaram menor sobrevida em comparação com os pacientes sem LRA (p < 0,05). O tempo mediano de seguimento foi de 22,6 meses. Dos 17 pacientes com doença renal crônica em estágio terminal, 11 (65%) evoluíram para óbito. Entre os 17 pacientes com LRA, 7 (41%) morreram durante o seguimento. Já entre os 77 pacientes sem LRA, 22 (29%) foram a óbito. NE = não avaliável.

Durante o período de seguimento, ocorreram 40 óbitos, correspondendo a uma incidência global de mortalidade de 36%. A mortalidade foi de 41% (7/17) entre pacientes com LRA, 65% (11/17) no grupo com DRT e 29% (22/77) entre pacientes sem LRA. Entre os pacientes que foram a óbito, a presença de DRC foi observada em 4 de 7 (57,1%) pacientes com LRA e em 10 de 22 (55,0%) pacientes sem LRA. Os dados sobre as causas de morte indicaram que os óbitos foram atribuídos principalmente a doença respiratória (26,3%), sendo 23,7% explicitamente relacionados à COVID-19, seguidos por causas cardiovasculares (18,4%) (Tabela Suplementar S1).

### Associação entre FGF23 e sobrevida global

As análises de regressão de *Cox* univariada e estratificada avaliando a associação entre FGF23 e sobrevida global estão apresentadas na Tabela 4. A cada aumento de dez anos na idade dos pacientes, a razão de risco (HR) não ajustada para óbito foi de 1,88 (IC 95%: [1,43–2,47]; p < 0,001). Para cada aumento de uma unidade em log(FGF23), a HR não ajustada para óbito foi de 1,42 (IC 95%: [1,18–1,71]; p < 0,001). Utilizando o grupo sem LRA como referência, a HR não ajustada para óbito foi de 1,52 (IC 95%: [0,65–3,56]; p = 0,30) entre pacientes com LRA e de 2,70 (IC 95%: [1,31–5,58]; p = 0,007) entre pacientes com DRT. Doença cardiovascular (HR = 3,40; IC 95%: [1,21–9,56]; p = 0,020) e IMC (HR = 0,79; IC 95%: [0,65–0,95]; p = 0,01) também apresentaram associação significativa com mortalidade. No modelo multivariável, as associações previamente observadas com doença cardiovascular e IMC deixaram de ser significativas (p > 0,05). Após ajuste para idade, doença cardiovascular e IMC, e considerando diferentes riscos basais de mortalidade entre os grupos com LRA e com DRT, log(FGF23) permaneceu significativamente associado a maior risco de óbito na coorte total (HR: 1,45; IC 95%: [1,02–2,07]; p = 0,04). Adicionalmente, em análise exploratória, não foi observada associação entre Klotho e LRA (p = 0,41) (Figuras Suplementares S2 e S3).

**Tabela 4:** Associação do FGF23 com maior mortalidade em pacientes com COVID-19.

Tipos de modelo	Variável	n	HR	IC 95%	Valor de p
Regressão de Cox univariada	Idade (aumento de 10 anos)	111	1,88	1,43–2,47	<0,001***
	Categoria de idade (ref: <65 anos)	111	7,30	2,85–18,7	<0,001***
	Hemoglobina (aumento de 1 g/dL)	104	0,94	0,79–1,12	0,47
	Diabetes (ref: não)	111	1,81	0,90–3,62	0,09
	Doença cardiovascular (ref: não)	111	3,40	1,21–9,56	0,02*
	IMC (aumento de 5 kg/m^2^)	101	0,79	0,65–0,95	0,01*
	log(FGF23) (aumento de 1 RU)	109	1,42	1,18–1,71	<0,001***
	Grupo LRA (ref: sem LRA) DRT (ref: sem LRA)	111	1,52 2,70	0,65–3,56 1,31–5,58	0,03*
Regressão de Cox multivariada estratificada	Idade (aumento de 10 anos)	99	1,81	1,31–2,51	<0,001***
	Doença cardiovascular (ref: não)		0,95	0,31–2,91	0,93
	IMC (aumento de 5 kg/m^2^)		0,86	0,69–1,09	0,21
	log(FGF23), (aumento de 1 RU)		1,45	1,02–2,07	0,04*

Abreviaturas – LRA: lesão renal aguda; IMC: índice de massa corporal; DRC: doença renal crônica; FGF23: fator de crescimento de fibroblastos 23; RU: unidades relativas.

Notas – Após ajuste para idade, histórico de doença cardiovascular e IMC, e considerando diferentes riscos basais de morte entre os grupos de LRA e DRC em estágio terminal, log(FGF23) manteve associação significativa com maior risco de morte em toda a coorte. Os asteriscos indicam níveis de significância: p < 0,05 (*),

p < 0,01 (**),

p < 0,001 (***).

Ausência de símbolo indica resultado não significativo (p ≥ 0,05).

## DISCUSSÃO

Até onde sabemos, este é o primeiro estudo a demonstrar que níveis circulantes elevados de FGF23 estão significativamente associados tanto à presença de LRA quanto à mortalidade em pacientes hospitalizados com COVID-19. A associação entre níveis elevados de FGF23 e a presença de LRA é particularmente relevante, considerando que a LRA é altamente prevalente em pacientes hospitalizados e está associada a elevada mortalidade, além de custos substanciais em saúde decorrentes de hospitalizações prolongadas^6^. Essas complicações podem ser agravadas pelo diagnóstico tardio de LRA. Dessa forma, a detecção precoce da LRA é fundamental, uma vez que pode facilitar intervenções oportunas e potencialmente melhorar os desfechos clínicos^15^. Nesse contexto, o uso de um biomarcador, como o FGF23, para avaliar o risco de desenvolvimento de LRA em pacientes com COVID-19 torna-se particularmente relevante, especialmente diante das elevadas taxas de mortalidade observadas entre pacientes hospitalizados ou admitidos em UTI^16^. O FGF23 circulante está presente em diferentes formas: uma forma intacta, biologicamente ativa, que pode ser clivada proteoliticamente em fragmentos N-terminal e C-terminal inativos^17^. Neste estudo, utilizamos um ensaio C-terminal de FGF23 capaz de detectar tanto as formas biologicamente ativas quanto as formas inativas da molécula.

Embora a incidência de LRA possa variar de acordo com os critérios utilizados e o contexto clínico (UTI vs. não UTI), estudos recentes na Europa e nos Estados Unidos sugerem que a incidência de LRA pode atingir até 20% entre pacientes hospitalizados e ultrapassar 50% entre pacientes com COVID-19 admitidos em UTI^18,19^. Em nossa coorte, observamos uma incidência ligeiramente menor de LRA (18%) entre pacientes com COVID-19. De forma semelhante, publicações recentes têm relatado considerável variabilidade na incidência de LRA entre pacientes hospitalizados com COVID-19. Por exemplo, Ng *et al*. observaram que, entre 9.657 pacientes hospitalizados com COVID-19, 40% desenvolveram LRA^3^. As diferenças na incidência de LRA entre pacientes com COVID-19 podem ser atribuídas a diversos fatores, incluindo a localização geográfica dos estudos, o período em que as observações foram realizadas e a gravidade da doença. Estudos provenientes da Ásia tendem a relatar menores taxas de LRA em comparação com aqueles conduzidos na Europa e nos Estados Unidos^18,19^. Essas diferenças provavelmente refletem variações nos critérios de hospitalização e no estado geral de saúde dos pacientes no momento da admissão. Em nosso estudo, observamos maior prevalência de comorbidades entre os pacientes com LRA, incluindo diabetes mellitus e doença cardiovascular. No grupo com LRA, 53% dos pacientes apresentavam diagnóstico prévio de DRC antes da admissão hospitalar, em comparação com 31% no grupo sem LRA. De forma semelhante, Ng et al.^3^ também demonstraram que pacientes com LRA apresentavam maior prevalência de comorbidades, como diabetes mellitus, doença arterial coronariana, insuficiência cardíaca e DRC.

O FGF23 foi inicialmente identificado como um importante regulador do metabolismo do fosfato, mas estudos subsequentes revelaram seu papel relevante em diversas vias metabólicas. Níveis elevados de FGF23 são comumente observados em pacientes com DRC, embora os mecanismos responsáveis por esse aumento ainda não estejam completamente esclarecidos^20^. Níveis elevados de FGF23 estão independentemente associados a maior risco de eventos cardiovasculares, progressão para DRT, perda precoce do enxerto após transplante e maior mortalidade entre pacientes com DRC^21,22^. Evidências emergentes também sugerem que os níveis de FGF23 se encontram elevados em pacientes com LRA, indicando seu potencial como biomarcador prognóstico^6,12^. Em concordância com esses achados, nossos resultados demonstram que os níveis de FGF23 foram significativamente mais elevados em pacientes com COVID-19 que apresentavam LRA ou DRT, em comparação com aqueles sem LRA. Adicionalmente, níveis elevados de FGF23 foram associados à presença de LRA e a maior mortalidade entre pacientes hospitalizados com COVID-19. Esses achados reforçam o potencial do FGF23 como biomarcador associado à presença de LRA e ao risco de mortalidade, o que pode contribuir para aprimorar a estratificação de risco e orientar estratégias de manejo clínico. Em contraste, os níveis de Klotho foram significativamente mais baixos em pacientes com DRT, sugerindo possível relação com a gravidade do comprometimento da função renal. Embora o Klotho tenha sido proposto como um potencial biomarcador precoce e alvo terapêutico para LRA^23^, em nosso estudo não foi observada associação entre os níveis de Klotho e a presença de LRA.

É particularmente interessante que evidências provenientes de estudos em modelos animais e em humanos sugiram que o aumento dos níveis de FGF23 após LRA precede alterações em outros biomarcadores clássicos de função renal. Isso inclui a lipocalina associada à gelatinase de neutrófilos (NGAL) em camundongos, bem como a creatinina sérica e outros metabólitos minerais em pacientes que desenvolveram LRA após cirurgia cardíaca ou em pacientes em estado crítico^6,12^. Estudos adicionais são necessários para melhor compreender como os níveis de FGF23 se comparam com outros biomarcadores de LRA, como o KIM-1. Além disso, o FGF23 tem emergido como um importante regulador envolvido em relações bidirecionais com anemia, metabolismo do ferro e inflamação, sugerindo seu possível papel na fisiopatologia da LRA^24,25^. Níveis elevados de FGF23 na LRA podem contribuir para agravar o ambiente inflamatório característico dessa condição. Como mediador inflamatório, o aumento do FGF23 pode intensificar o dano renal ao promover calcificação vascular e amplificar a resposta inflamatória^26^. Esse processo pode estabelecer um ciclo de retroalimentação no qual a inflamação agrava a LRA, elevando ainda mais os níveis de FGF23 e perpetuando a lesão renal^24^. Além disso, estudos recentes têm destacado os efeitos adversos do FGF23 sobre o metabolismo do ferro^25,27^. Níveis elevados de FGF23 estimulam a produção de hepcidina, reduzindo a absorção intestinal de ferro e os níveis circulantes desse mineral. Por outro lado, a deficiência de ferro ou a presença de anemia pode aumentar os níveis de FGF23, estabelecendo um ciclo de retroalimentação potencialmente deletério. Essas interações podem comprometer a recuperação renal após lesão aguda, afetando negativamente a eritropoiese e o metabolismo sistêmico^25^. Outros mecanismos potencialmente envolvidos incluem o papel de níveis elevados de FGF23 na indução de sinais profibróticos em fibroblastos renais ativados pela lesão, o que pode contribuir para a progressão do dano renal além do insulto inicial. Além disso, o FGF23 pode aumentar a atividade de miofibroblastos durante a LRA, possivelmente promovendo a cascata de sinalização que culmina em fibrose renal. Níveis plasmáticos mais elevados de FGF23 também têm sido associados a maior risco de infecção, possivelmente por meio da modulação da resposta imune inata. Dessa forma, o FGF23 pode estar envolvido na progressão da LRA no contexto da infecção por COVID-19, embora essa hipótese não tenha sido diretamente investigada no presente estudo. Em conjunto, esses achados ressaltam a complexidade dos efeitos adversos associados a níveis elevados de FGF23 e seus impactos na saúde renal.

Nossa coorte apresentou uma taxa de mortalidade de 41% entre pacientes com LRA, o que está em consonância com achados semelhantes descritos em outros estudos. A disfunção orgânica, envolvendo particularmente os pulmões e os rins, constitui um importante indicador de gravidade da COVID-19 e está associada a taxas de mortalidade significativamente elevadas^28^. A LRA é considerada um marcador prognóstico negativo para a sobrevida, especialmente entre pacientes internados em UTI^29^. Além disso, pacientes com DRT apresentam sintomas mais graves e maior risco de mortalidade por COVID-19^30^. De forma consistente com estudos prévios, observamos uma taxa de mortalidade mais elevada (65%) em pacientes com DRT em nossa coorte. No entanto, na Europa, pacientes com DRT e COVID-19 têm apresentado taxas de mortalidade variando entre 20% e 30%^27^. De forma semelhante, a variabilidade observada nas taxas de incidência também se reflete nas taxas de mortalidade. Essa divergência pode ser atribuída a diversos fatores, incluindo tempo prolongado de hospitalização, uma população amostral numerosa e racial ou etnicamente diversa, além de diferenças no acesso à saúde e na alocação de recursos. Esses achados preocupantes ressaltam a necessidade urgente de estratégias para avaliar o risco de LRA associada à infecção por COVID-19. A detecção precoce da LRA, aliada a medidas terapêuticas adequadas, é fundamental para mitigar desfechos adversos e reduzir a elevada taxa de mortalidade entre pacientes com COVID-19.

Os resultados deste estudo devem ser interpretados considerando seus pontos fortes e limitações. Entre os principais pontos fortes, destacamos que este é o primeiro estudo prospectivo e longitudinal a avaliar os níveis de FGF23 em pacientes com COVID-19 em relação a desfechos clinicamente relevantes, incluindo LRA e mortalidade. Outros aspectos relevantes incluem uma coorte bem caracterizada e um delineamento que contempla três grupos: pacientes sem LRA, pacientes com LRA e indivíduos com DRT. Essa abordagem permite uma avaliação abrangente do papel do FGF23 em diferentes graus de comprometimento renal, conferindo maior robustez aos achados. O estudo também apresenta limitações importantes. A principal refere-se ao tamanho reduzido da amostra, limitado pela disponibilidade de pacientes no *Indiana Biobank*. Além disso, as concentrações de FGF23 foram medidas apenas no momento basal, em razão da ausência de amostras seriadas, o que restringe a avaliação de alterações ao longo do tempo e sua relação com a progressão da LRA. Adicionalmente, o uso de corticosteroides e de ventilação mecânica – frequentes no manejo da COVID-19 em 2020 – não foi capturado de forma consistente no banco de dados e, portanto, não pôde ser incluído nas análises multivariadas. Por fim, não houve acesso a desfechos intermediários adicionais, como dados ecocardiográficos, que poderiam fornecer informações relevantes sobre desfechos relacionados à função renal e cardiovascular.

Em conclusão, nossos achados demonstram que níveis elevados de FGF23 circulante estão significativamente associados à presença de LRA e a uma maior mortalidade em pacientes hospitalizados com COVID-19. Especificamente, os níveis de FGF23 foram substancialmente mais elevados em pacientes com LRA e com DRT, em comparação com aqueles sem LRA. Esses resultados reforçam o potencial do FGF23 como biomarcador relevante na avaliação do risco de LRA e mortalidade em pacientes com COVID-19, podendo contribuir para o aprimoramento das estratégias de estratificação de risco e manejo clínico. Além disso, estudos adicionais são necessários para aprofundar a compreensão do papel do FGF23 na tomada de decisão clínica e na melhoria dos desfechos em pacientes em risco de LRA, incluindo sua possível associação com a progressão subsequente da DRC, bem como os mecanismos envolvidos na fisiopatologia da LRA.

## Disponibilidade de dados

Os dados gerados e/ou analisados durante o presente estudo não estão disponíveis publicamente devido a restrições de privacidade, mas podem ser disponibilizados pelo autor correspondente mediante solicitação razoável.

## MATERIAL SUPLEMENTAR

O seguinte material online está disponível para o presente artigo:

- Tabela S1 - Distribuição das principais causas de óbito (n = 40).
- Figura S1 - Correlação entre FGF23 e idade em pacientes com COVID-19.
- Figura S2 - Níveis de Klotho de acordo com o *status* de LRA em pacientes com COVID-19.
- Figura S3 - A análise de *Kaplan-Meier* não demonstrou diferença significativa na probabilidade de sobrevida entre pacientes com COVID-19 agrupados de acordo com os níveis de Klotho e o *status* de LRA.
